# Brain Networks Involved in Sensory Perception in Parkinson’s Disease: A Scoping Review

**DOI:** 10.3390/brainsci13111552

**Published:** 2023-11-06

**Authors:** Fiona Permezel, Jane Alty, Ian H. Harding, Dominic Thyagarajan

**Affiliations:** 1 Department of Neuroscience, Monash University, Melbourne 3004, Australia; fiona.permezel@monash.edu (F.P.); ian.harding@monash.edu (I.H.H.); 2 Department of Neurology, Mayo Clinic, Rochester, MN 55901, USA; 3 Wicking Dementia Research and Education Centre, University of Tasmania, Hobart 7001, Australia; jane.alty@utas.edu.au

**Keywords:** Parkinson’s, basal ganglia, sensory, sensorimotor, brain network, microelectrode, functional magnetic imaging, positron emission tomography

## Abstract

Parkinson’s Disease (PD) has historically been considered a disorder of motor dysfunction. However, a growing number of studies have demonstrated sensory abnormalities in PD across the modalities of proprioceptive, tactile, visual, auditory and temporal perception. A better understanding of these may inform future drug and neuromodulation therapy. We analysed these studies using a scoping review. In total, 101 studies comprising 2853 human participants (88 studies) and 125 animals (13 studies), published between 1982 and 2022, were included. These highlighted the importance of the basal ganglia in sensory perception across all modalities, with an additional role for the integration of multiple simultaneous sensation types. Numerous studies concluded that sensory abnormalities in PD result from increased noise in the basal ganglia and increased neuronal receptive field size. There is evidence that sensory changes in PD and impaired sensorimotor integration may contribute to motor abnormalities.

## 1. Introduction

Parkinson’s disease (PD) was first described by James Parkinson over 200 years ago [1] and is diagnosed by ascertaining the presence of movement abnormalities, namely bradykinesia and at least one additional symptom: tremor, muscle rigidity, or postural/balance instability not attributed to primary visual, cerebellar, or proprioceptive dysfunction [2]. The core pathology is believed to originate in the basal ganglia, which is crucial for motor function, beginning with the loss of dopamine-secreting neurons in the substantia nigra (SN) pars compacta. This in turn leads to significant changes in other basal ganglia nuclei, resulting in excessive inhibition of thalamocortical and brainstem motor systems [3]. Application of deep brain stimulation (DBS) to suppress globus pallidus interna (GPi) or subthalamic nucleus (STN) activity has been demonstrated to alleviate PD motor symptoms and enhance quality of life [4].

People with Parkinson’s Disease (PwPD) typically experience a decline in independence in daily activities around 7 years post-diagnosis [5]. This initial decline in function is not attributed to a loss of motor response to levodopa, nor motor fluctuations [6], but may be at least partially ascribed to non-motor mechanisms that gradually accumulate and contribute to the burden of disease over time.

The pathology in PD has historically been associated predominantly with motor domain dysfunction; however, there is a growing body of evidence that abnormalities in sensory function also occur e.g., [7,8,9,10,11,12]. Sensory abnormalities occur across a range of modalities, including proprioceptive, tactile, visual, auditory and temporal perception functions. These contribute directly to functional disability in PwPD [13]. Furthermore, sensory dysfunction in PD may also account for a component of the motor impairment via the process of sensorimotor integration, by which sensory stimuli integrate with motor circuits within the brain to enable sensory-regulated control of motor responses (e.g., [14]). Additionally, the process of sensorimotor integration in PD is impaired (e.g., [15]), which may add further to motor impairment. Sensory abnormalities impacting motor function may therefore indirectly further contribute to the burden of disease [16].

Recent advances suggest that neuromodulation of the basal ganglia can correct not only motor, but also certain sensory abnormalities in PD [8,17,18]. Elucidating the neural substrates of sensory abnormalities in PD may therefore suggest potential targets for surgical neurointervention as a means to improve sensory disturbance in PD. This information may also provide greater understanding around sensory side effects that result from basal ganglia neurostimulation. These avenues of inquiry can also uncover new treatment options for this aspect of PD disability. While evidence for the importance of the basal ganglia in sensory processing is growing, there has not previously been a summary of these studies, nor a review of this literature relating to the abnormalities of sensory processing in PD.

This scoping review therefore aims to summarise research on sensory abnormalities in PD and identify current knowledge gaps through addressing the following research questions: (i) What brain areas are involved in sensory processing? (ii) What are the abnormalities in PD brain function during sensory processing? (iii) What is the importance of the basal ganglia in sensory processing? (iv) What could be the neuropathological basis behind the reduced sensory function in PD? (v) How does the brain integrate sensory and motor function and what is the significance of this with regards to PD?

## 2. Methods

### 2.1. Search Strategy

A comprehensive search strategy, aiming to address the outlined research questions, was employed utilizing the MEDLINE database, focusing on English language publications. Various terms related to Parkinson’s disease, sensory functions, and related methodologies were included, such as “parkinson*”, “somatosensory”, “sensorimotor”, “sensation”, “DBS”, “stimulation”, “basal ganglia”, “fMRI”, “PET”, among others. For a detailed list of search terms and the complete search strategy, refer to the Supplementary Materials.

The search encompassed literature from the inception of MEDLINE until 27 June 2022. We included original research publications without bias consideration or sample size restriction, focusing on studies exploring brain sensory function in humans with PD, healthy humans, healthy animals and animal models of PD. Studies using various brain function interrogation modalities like functional magnetic resonance imaging (fMRI), positron emission tomography (PET), transcranial magnetic stimulation (TMS), theta band stimulation or microelectrode recordings (MER) were considered. Excluded were review articles, case reports, abstracts, and meta-analyses. There was no date restriction. For a comprehensive overview of inclusion and exclusion criteria and the full search strategy, see the Supplementary Materials.

This review, adhering to PRISMA guidelines [19], was facilitated by Covidence software. Each article underwent independent screening by title and abstract by two researchers (FP and one of DT, IH, and JA), with conflicts resolved through discussion or a third researcher’s decision.

### 2.2. Data Extraction

Data extraction and full-text review were conducted by one researcher (FP). Extracted data included sample size, comparison condition/s, animal model of PD, PD disease severity, sensory type, sensory test details, brain activity localizing test, brain stimulation/suppression tool, sensory test outcome, basal ganglia findings, cortical findings, changes with antiparkinsonian medication and changes with DBS, where applicable.

Summary images of activated brain regions were generated solely from studies that controlled for confounding by motor activation or other sensory modalities. Only brain areas activated by a sensory stimulus and replicated across two or more studies were included in the summary images. An exception was made when no brain areas for a particular sensory stimulus were found to be activated across multiple studies; in such cases, areas activated by a particular sensory stimulus in a single study were included in the summary images.

## 3. Results

### 3.1. Study Selection

**Figure 1:**
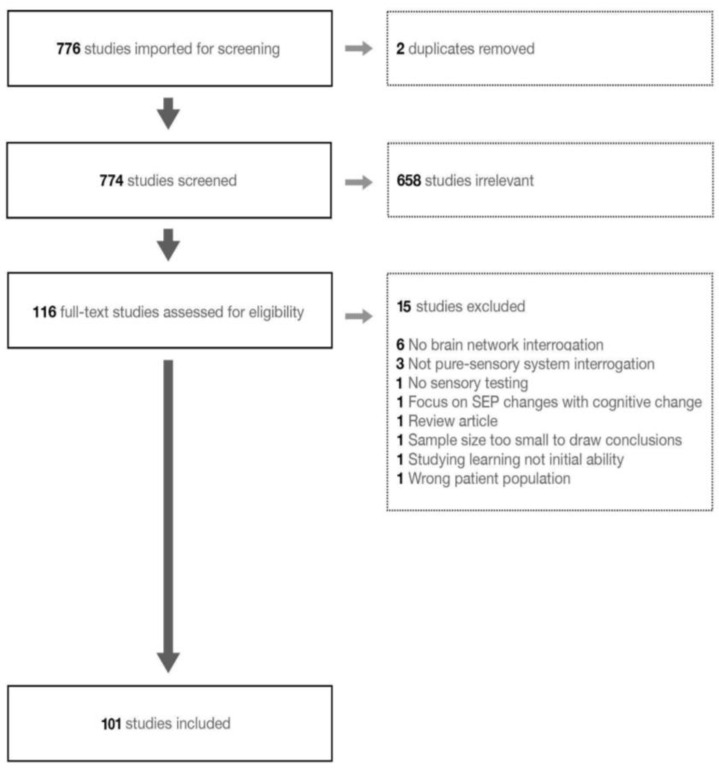
PRISMA scheme of reviewed literature.

The implemented search strategy yielded 776 studies for screening (Figure 1), 116 of which met the inclusion criteria. After full-text review, 101 articles were selected for inclusion in the review, all being cross-sectional in nature.

### 3.2. Sample Characteristics

#### 3.2.1. Breakdown of Sample Data

**Figure 2:**
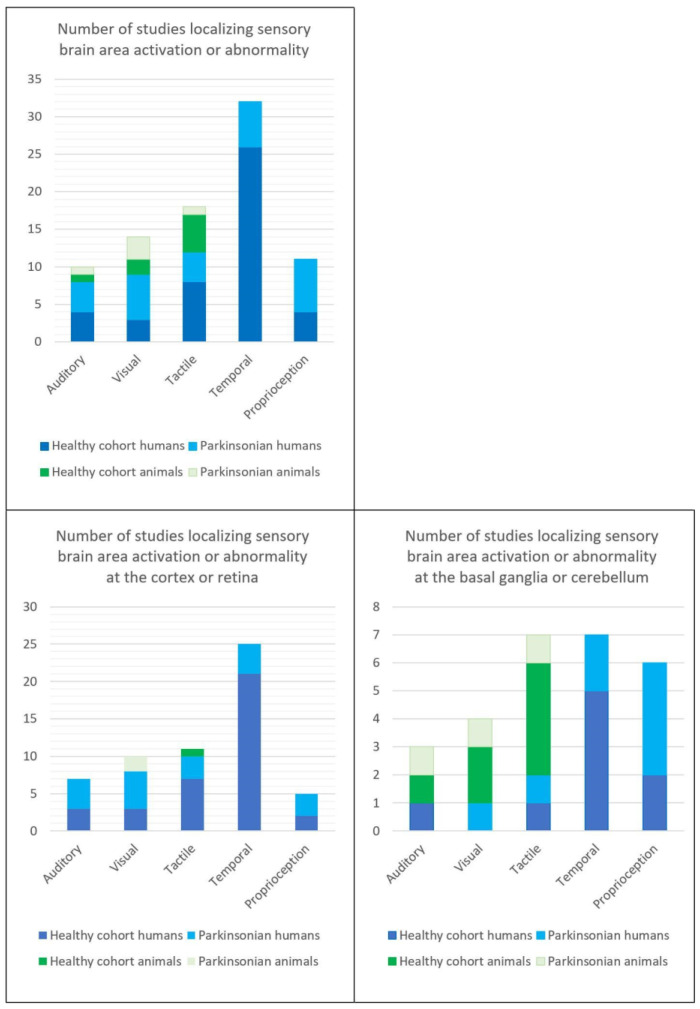
Breakdown of the numbers of studies extracted between disease state, animal or human cohort and sensory modality.

**Figure 3:**
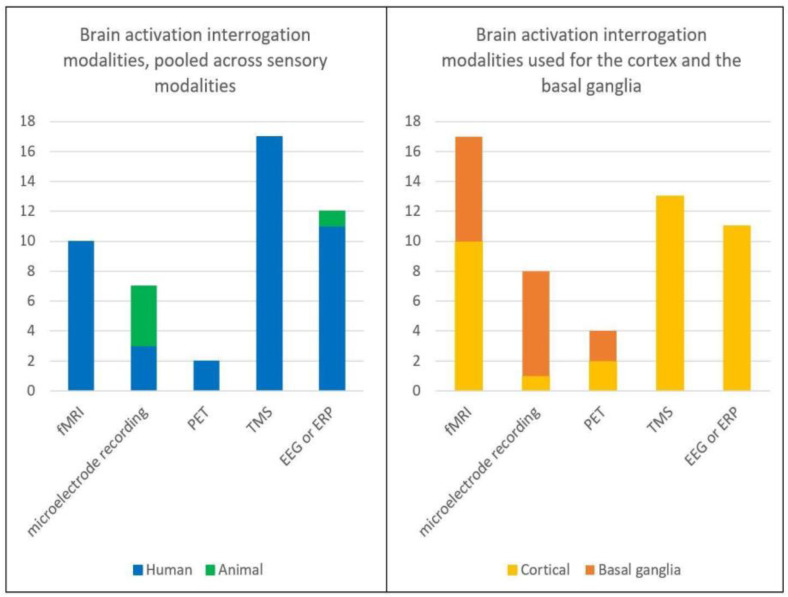
Breakdown brain activation interrogation modalities. fMRI = functional magnetic resonance imaging, PET = positron emission tomography, TMS = transcranial magnetic stimulation, EEG = electroencephalogram, ERP = event related potential.

The included articles comprised 89 human and 12 animal studies, published between 1982 and 2022. Animal studies included rats, cats, and monkeys. In total, 2853 human subjects and 125 animals were studied. Specific details on the divisions between healthy control (HC) vs. PD, animals vs. human, and the brain interrogation modality used are reported in Figure 2 and Figure 3, and the Supplementary Materials.

Forty-eight studies evaluated the brain areas activated during sensory stimulation. Only one study assessed multisensory integration [20]. Thirty studies investigated evoked potentials and were not included in the assessment of localization of sensory response due to the mixed proprioceptive–tactile nature of the stimulus. Thirteen studies assessed the somatotopic organisation of the basal ganglia. Seven studies evaluated sensorimotor integration and nine studies investigated increased noise or decreased neuronal activation specificity in the basal ganglia in PD. See the Supplementary Materials for further details of the sample characteristics.

In the review of the literature presented below, studies were undertaken in humans unless otherwise specified.

#### 3.2.2. Sensory Testing Methodologies

Human sensory testing methods were as follows:

(i) Temporal sensory-system testing utilized temporal (tactile and auditory) discrimination thresholds, temporal order judgement, temporal (auditory and visual) duration discrimination and time reproduction tasks.
(ii) Auditory sensory-system testing utilized frequency discrimination (deviant tones) and spatial discrimination (localizing site of tone).
(iii) Visual sensory-system testing utilized light intensity discrimination, pattern (moving or static) discrimination, shape recognition, face (emotion and gender) recognition and flickering checkerboard.
(iv) Tactile system testing utilized tactile shape discrimination, tactile roughness discrimination, tactile grating orientation discrimination, tactile amplitude (of ring electrode impulse) discrimination, tactile spatial discrimination (defining site of arm stimulated), tactile vibratory-frequency discrimination.
(v) Proprioception was stimulated with passive movement of a joint; however, the acuity of proprioception was not tested in the studies which were identified by our search strategy.

Animal sensory systems were stimulated with light spots on a high contrast background, moving vertical gratings (visual), monaural clicks or white noise (auditory), whisker deflection, light touch to shaved skin, brushing of hair, punctate stimulation (tactile), passive joint movement (proprioception) and muscle palpation/tendon tap (mixed tactile and proprioception).

### 3.3. What Brain Areas Are Involved in Sensory Processing?

We present the evidence from the literature for each sensory modality, as follows: temporal processing, auditory, visual, tactile and proprioception. The findings are sub-categorised as evidence from healthy controls (HC) first and then from PwPD.

#### 3.3.1. What Brain Areas Are Involved in Temporal Processing in Healthy Cohorts?

**Figure 4:**
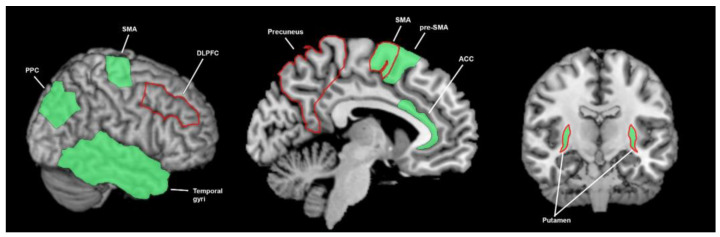
Brain areas involved in temporal processing; the green shading denotes areas found to be involved in healthy controls (ACC = 1 study, SMA = 4 studies, pre-SMA = 3 studies, temporal gyri = 3 studies, PPC = 3 studies, putamen = 4 studies) and the red outline denotes areas found to function abnormally in patients with Parkinson’s disease (DLPFC = 1 study, SMA = 1 study, precuneus = 1 study, putamen = 1 study). This summarises findings from 13 human studies. PPC = posterior parietal cortex, SMA = supplementary motor area, DLPFC = dorsolateral prefrontal cortex, SMA = supplementary motor area, pre-SMA = pre supplementary motor area, ACC = anterior cingulate cortex.

The putamen, posterior parietal cortex (PPC), supplementary motor area (SMA), temporal gyri, pre-SMA and anterior cingulate cortex (ACC) are active in temporal perception (the perception of the magnitude of time interval) (Figure 4). The SMA is involved in tactile [21,22], visual [23] and auditory [24] forms of temporal discrimination. The pre-supplementary motor area (pre-SMA) is shown to be activated in tactile [21,25] and auditory [26] forms of temporal discrimination. The temporal lobe (including the superior temporal gyrus and temporal gyri) is involved in tactile [21,27] and visual [23] forms of temporal discrimination. The PPC is involved in visual and auditory [28] forms of temporal discrimination. The PPC is also involved in tactile forms of temporal discrimination [22,29] although neither of these studies controlled for brain-area activation due to the tactile aspect of the response. However, in light of the Bueti et al. [28] finding that found PPC involvement during temporal discrimination assessed by two different sensory modalities (visual and auditory), these results are likely reflective of temporal processing.

All of these areas demonstrate bilateral involvement in temporal processing [21,23,24,25,29], although some studies have shown that only the ipsilateral or contralateral side was active [21,26,27,28].

The putamen is also involved in temporal discrimination [21,22,30] and duration discrimination [23]. Additionally, the putamen is involved in temporal processing in conjunction with auditory [30], visual [23] and tactile [21] stimulation. Nenadic [30], Ferandez [23] and Huang [21] compared temporal with other types of discrimination in one sensory modality, and found that the putamen was specifically involved in temporal processing.

#### 3.3.2. What Brain Areas Are Found to Function Abnormally in Temporal Processing in People with Parkinson’s Disease?

Koch [31] found that repetitive TMS applied to the dorsolateral prefrontal cortex (DLPFC) could improve time perception in PD but not HCs (Figure 4). Repetitive TMS to the SMA had no significant effect for PwPD or controls. Elsinger [24] found decreased SMA activity during a pace-continuation condition in PwPD off medication, but not on medication, when compared to HCs. The pace-continuation condition required patients to continue tapping their finger at a pace previously demonstrated with auditory tones after cessation of the auditory guide. Dusek [32] found that PwPD on medication, compared to PwPD off medication, had increased bilateral precuneus activation during retrieval of a remembered interval duration (temporal estimation), which correlated with a trend to improved performance in PwPD when on medication, suggesting the importance of the precuneus in temporal estimation in PD. Elsinger [24] also found decreased putamen activity in PwPD off medication, compared to HCs, during a pace-continuation condition that may (as mentioned above) either reflect pure temporal processing or memory retrieval.

Studies exploring brain connectivity in non-PD cohorts have provided evidence of a network of temporal perception, which may help identify a neuropathological substrate underlying abnormal temporal perception in PD cohorts. Lacruz [22] noted functional impacts of lesion locations in the brain and through this noted the main cortical output from the basal ganglia is to the SMA. The SMA then projects heavily to the superior parietal lobe, with the SMA [21,22,23,24] and PPC [22,28,29] noted to be involved in temporal processing. In a healthy cohort study, Pastor [25] noted that the pre-SMA receives input from the basal ganglia and cerebellum and sends output to the striatum and STN. Also in a healthy cohort, Huang [21] noted that ability in the somatosensory temporal-discrimination task was predicted not only by activity in the pre-SMA, ACC and dorsal putamen, but by the connection between these areas. The reduced temporal-processing ability in PD [24,31,33,34], the diseased state of the putamen [35] in PD and the abnormal SMA and putaminal activity [24] noted during temporal-perception testing in a PD cohort, provide further evidence for this putamen, SMA, pre-SMA and ACC network of temporal perception.

#### 3.3.3. What Brain Areas Are Involved in Auditory Processing in Healthy Cohorts?

**Figure 5:**
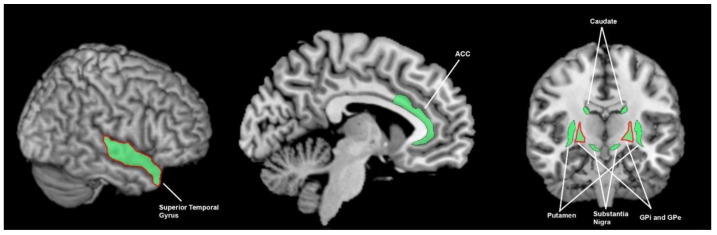
Brain areas involved in auditory processing; the green shading denotes areas found to be involved in healthy controls (ACC = 2 studies, superior temporal gyrus = 2 studies, putamen = 1 study, globus pallidus = 1 study, SN = 1 study, caudate = 1 study) and the red outline denotes areas found to function abnormally in patients with Parkinson’s disease (superior temporal gyrus = 3 studies, globus pallidus = 1 study). This summarises findings from 7 human studies and 2 animal studies. ACC = anterior cingulate cortex. GPi = globus pallidus internal, GPe = globus pallidus externa.

Multiple studies have found that the bilateral superior temporal gyri [24,30] and ACC (left or right) [26,30] are important in auditory processing (Figure 5). Pastor [26] found the right head of caudate and putamen are active in both auditory spatial and auditory temporal discrimination. This finding is supported by the animal study by Nagy [20] that found 6% of neurons in the caudate tested in cats responded to auditory stimuli on MER. Nagy et al. also found that SN neurons also respond to auditory stimuli. Rothblat [36] found, via microelectric recording in cats, that 22% of globus pallidus externa (GPe) and 8% of GPi neurons responded to auditory stimulation.

#### 3.3.4. What Brain Areas Are Found to Function Abnormally during Auditory Processing in Patients with Parkinson’s Disease?

Pekkonen [37] and Philipova [38] found a reduced amplitude N1 and frontal mismatch negativity (MMN) or P3 response on an electroencephalogram (EEG) during auditory frequency discrimination in PwPD (Figure 5). The N1 response is thought to originate in the superior temporal auditory cortex during auditory stimulus detection. The frontal MMN and P3 responses are thought to reflect auditory stimulus evaluation and discrimination. Rossi [39] found reduced amplitude and prolonged latencies of auditory evoked potentials in PwPD, when compared to HCs, but Weise [40] found no difference. With regards to basal ganglia changes in PwPD in auditory processing, Rothblat [36] found on MER that GPe response to auditory stimulation reduced from 22% to 5%, and GPi response reduced from 8% to 4%.

#### 3.3.5. What Brain Areas Are Involved in Visual Processing in Healthy Cohorts?

**Figure 6:**
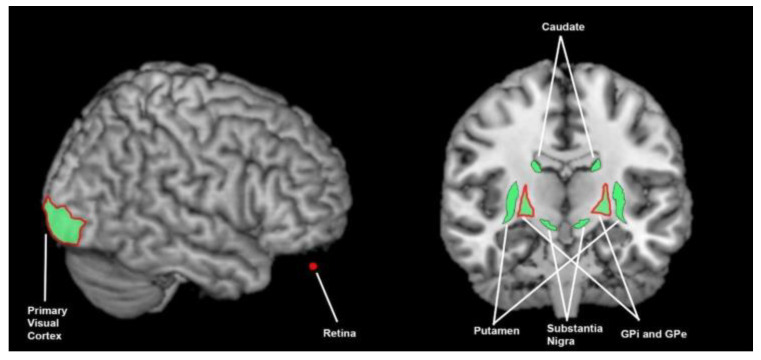
Brain areas involved in visual processing; the green shading denotes areas found to be involved in healthy controls (primary visual cortex = 2 studies, caudate = 2 studies, putamen = 1 study, substantia nigra = 2 studies, globus pallidus = 1 study) and the red outline denotes areas found to function abnormally in patients with Parkinson’s disease (primary visual cortex = 4 studies, retina = 4 studies, globus pallidus = 1 study). This summarises findings from 8 human studies and 2 animal studies. GPi = globus pallidus interna, GPe = globus pallidus externa.terior cingulate cortex. GPi = globus pallidus internal, GPe = globus pallidus externa.

Several studies found occipital activity during visual discrimination tasks [23,41] (Figure 6). Schmiedt et al. additionally found when assessing the event-related potential response that occipital excitability was early, and likely related to low-level visual processing. Ferrandez also found evidence of caudate, putamen and SN/red nucleus involvement on fMRI during visual discrimination tasks. Ferrandez hypothesized that the putamen and caudate were active due to the demand of making sequential decisions, and the SN/red nucleus was responsible for linking other important structures together. However, animal studies suggest that the basal ganglia activation may be directly induced by visual stimuli. Nagy [20] found in MER in cats that 17% of caudate neurons tested responded to visual (light) stimuli. There was a similar pattern in the SN. Rothblat [36] found 16% of GPe equivalent neurons to respond to visual light stimulation, also via MER in cats, and 11% of GPi equivalent neurons to do the same.

#### 3.3.6. What Brain Areas Are Found to Function Abnormally during Visual Processing in People with Parkinson’s Disease?

During visual stimulation tasks, there is reduced visual cortex activity in PD compared to HCs [42,43,44,45] (Figure 6). There is additionally pattern electroretinogram evidence of retinal dysfunction during visual stimulus in PD [42,44,46,47]. Rothblat [36] additionally demonstrated reduced GPi and GPe microelectrode responses to visual stimuli in an 1-methyl-4-phenyl-propionoxy-piperidine (MPTP) model of PD in cats.

#### 3.3.7. What Brain Areas Are Involved in Tactile Processing in Healthy Cohorts?

**Figure 7:**
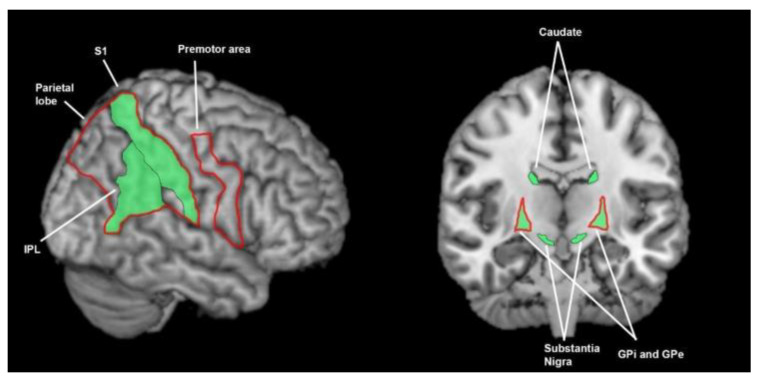
Brain areas involved in tactile processing; the green shading denotes areas found to be involved in healthy controls (S1 = 7 studies, IPL = 1 study, caudate = 3 studies, substantia nigra = 2 studies, globus pallidus = 2 studies) and the red outline denotes areas found to function abnormally in patients with Parkinson’s disease (S1 = 3 studies, parietal lobe = 2 studies, premotor area = 2 studies, globus pallidus = 1 study). This summarises findings from 7 human and 4 animal studies. IPL = inferior parietal lobe, S1 = primary somatosensory cortex, GPi = globus pallidus interna, GPe = globus pallidus externa.

Unsurprisingly, a number of studies have demonstrated the importance of the contralateral primary sensory cortex (S1) in tactile sensation processing [25,27,48,49,50] (Figure 7). Mowery [51] also demonstrated the importance of the contralateral S1 in a non-PD animal model with MER during whisker deflection in rats. Pastor additionally found S1 and the inferior parietal lobule (IPL) activation common to both tactile spatial and tactile temporal discrimination. The work of Zhao [52] in HCs further supports the importance of bilateral S1 and contralateral IPL (Brodmann area 40) in tactile sensory perception.

Pastor [25] found bilateral basal ganglia nuclei activity in both tactile spatial and tactile temporal discrimination, including the caudate and SN. This is supported by animal models looking at MER response to tactile stimulation in the basal ganglia. Mowery [51] and Nagy [20] found caudate responsiveness to tactile stimuli. Nagy also noted a similar response in the SN. The DeLong [53] and Rothblat [36] animal studies also noted neuronal responses in the GPi and GPe to tactile stimulation.

#### 3.3.8. What Brain Areas Are Found to Function Abnormally during Tactile Processing in Patients with Parkinson’s Disease?

Weder [54] and Zhao [52] revealed reduced contralateral S1, bilateral parietal lobe and contralateral or bilateral premotor area activity during tactile spatial discrimination tasks (Figure 7). These findings were supported by Palomar [48] who was able to improve tactile amplitude discrimination with paired-pulse transcranial magnetic stimulation (ppTMS) to the contralateral S1 in HCs, but the same benefit was not found in PwPD on medication. The finding was not significant in patients off medication. In animal study MER, Rothblat [36] demonstrated decreased globus pallidus neuronal function to tactile stimuli, as GPe-equivalent neurons reduced from 31.4 to 12.2% response, and GPi-equivalent neurons reduced from 29% to 13% response.

#### 3.3.9. What Brain Areas Are Involved in Proprioceptive Processing in Healthy Cohorts?

**Figure 8:**
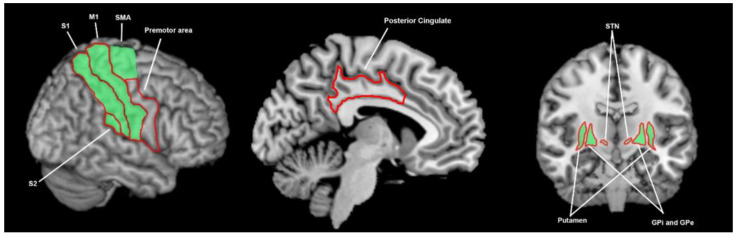
Brain areas involved in proprioceptive processing; the green shading denotes areas found to be involved in healthy controls (S1 = 2 studies, S2 = 1 study, M1 = 1 study, SMA = 1 study, putamen = 1 study, globus pallidus = 3 studies, STN = 2 studies) and the red shading denotes areas found to function abnormally in patients with Parkinson’s disease (S1 = 2 studies, S2 = 1 study, M1 = 1 study, lateral premotor = 1 study, posterior cingulate = 1 study, STN = 1 study, globus pallidus = 1 study, putamen = 1 study)**.** This summarises findings from 12 human and 2 animal studies. S2 = secondary somatosensory cortex, S1 = primary somatosensory cortex, M1 = primary motor cortex, SMA = supplementary motor area, STN = subthalamic nucleus, GPi = globus pallidus interna, GPe = globus pallidus externa.

Kalmar [35] and Boecker [55] demonstrated contralateral S1 activity during proprioceptive stimulation, with Boecker showing this extended to the contralateral S2, and Kalmar also revealing activity in the contralateral primary motor cortex (M1) and SMA (Figure 8). In an HC population, Kalmar found the contralateral putamen to activate on fMRI in response to proprioceptive stimuli, while the Boecker study revealed contralateral globus pallidus activation. Non-PD animal studies [53,56] have revealed neuronal activation on MER in the GPe, GPi, and STN in response to proprioceptive stimuli of the face, arm and leg.

#### 3.3.10. What Brain Areas Are Found to Function Abnormally during Proprioceptive Processing in Patients with Parkinson’s Disease?

Seiss [57] found an early evoked EEG response to proprioception, attributed to a focus in the primary motor cortex, was equivalent between PwPD and HCs, but a later response, attributed to a source in the S1 cortex, was of opposite polarity in PwPD. This is supported by Boecker’s [55] findings that the contralateral S1 had decreased activity in PD patients compared with controls (Figure 8), although Boekcer also found that the contralateral M1, lateral premotor, S2 and posterior cingulate had decreased activity in PwPD.

A number of studies have demonstrated that the GP [58,59], STN [60,61,62,63,64] and pedunculopontine nucleus [65] are responsive to proprioception in PD cohorts, although without comparison to HCs to assess for effects of the disease state. Stefani [66] demonstrated in PwPD that there is an increase in the contralateral STN neuronal firing to passive movement, with this firing rate reduced by apomorphine. Boecker [55] found the contralateral GP to have decreased activation in a PD cohort during proprioceptive stimulation, with the contralateral putamen demonstrating a trend towards the same effect. Kalmar [35] demonstrated an increase in ipsilateral putamen activity only in right-handed PwPD, which may suggest a diseased state of the contralateral putamen.

### 3.4. The Breadth of Sensory Response and Multisensory Response of the Basal Ganglia

DeLong [53] looked at MER in HC monkeys in the GPe, GPi and STN after passive joint movement (proprioception > tactile), joint palpation (tactile and possibly proprioception), muscle palpation (tactile and possibly proprioception), tendon taps (tactile and proprioception), body hair stimulation (thought to be purely tactile stimulation) and light touch (tactile stimulation alone). All of the tested basal ganglia nuclei had by far the greatest activity during passive joint movement, which may be thought as the closest representation of proprioception. In the same study, MER revealed no response to a light touch, supporting an argument that the basal ganglia specifically detect proprioception, and indirectly support motor function through this mechanism. Low-magnitude responses in these nuclei were recorded during joint palpation, muscle palpation and tendon taps. This response may reflect low-magnitude proprioceptive responses with these forms of stimulation. Similarly in HC humans, Kamar [35] demonstrated putaminal fMRI activation during passive joint movement and Boecker [55] demonstrated globus pallidus PET activation to high-frequency vibrational stimulus to a joint. There are additionally numerous studies investigating proprioceptive responses in the basal ganglia with MER in PD humans, which were conducted in the setting of DBS surgeries. Stefani [66] found contralateral STN activation on MER in PD humans during proprioceptive stimuli. Additionally, there are many studies investigating the somatotopic organization of the STN in PD humans to proprioceptive stimuli [58,59,60,61,62,63,64,67,68,69]. The low-magnitude activation during hair stimulation reported by DeLong and colleagues [53], however, is difficult to rationalize as the result of activation of proprioceptive afferents, and therefore this finding may argue towards the ability of the basal ganglia to also detect non-proprioceptive sensations.

Pastor [25] demonstrated with fMRI a tactile response at the head of caudate, STN and SN, and an auditory response at the head of caudate and putamen in human HCs. Additionally, studies have demonstrated basal ganglia responses to various sensory stimuli via MER in HC animals [20,36,51,53], supportive of the notion that the basal ganglia are involved in non-proprioceptive sensory perception (hair stimulation, visual, tactile, auditory stimuli). Pesenti [70], in a human study, however, failed to detect any component of the P100 visual-evoked potential response via MER at the STN in a PD cohort, although did not include a comparison to HCs leaving it unclear if this was due to the impact of PD or merely a lack of STN activity with visual stimulus. Only one study demonstrated the multisensory response of basal ganglia neurons. Nagy [20] examined neuronal responses in the caudate and SN to visual, auditory and tactile stimulation in HC cats. Fascinatingly, Nagy found that in the caudate, more neurons responded to more than one sensory modality than to a single modality. This single neuronal response to multisensory modalities was greater than the additive response of the different modalities alone, suggesting a preference of these neurons for a multisensory response. In addition, latencies to multimodal sensory responses were actually shorter than to unimodal responses. The SN also has a multi-sensory function, although with a higher proportion of neurons responding to one modality only. Rothblat et al. [36] also provided some insight into changes that may occur to the multi-sensory response in the basal ganglia in PD. They found, in an MPTP model of PD in cats, there was increased multi-sensory response in the GPe and GPi, in that more neurons responded to multiple sensory types than before the induction of Parkinsonism.

## 4. Discussion

This review synthesizes findings from 101 studies conducted between 1982 and 2022, involving 2853 humans and 137 animals, addressing key questions regarding sensory processing abnormalities in PD.

### 4.1. What Brain Areas Are Involved in Sensory Processing, What Are the Abnormalities in Parkinson’s Disease, and What Is the Importance of the Basal Ganglia?

In observing the active brain areas in the visual, auditory, tactile, proprioceptive and temporal senses, and the abnormalities in PD, a hypothesis emerges that PwPD demonstrate sensory-processing abnormalities due to abnormal functioning of the basal ganglia. Amongst tactile, auditory, visual and temporal perception and proprioception, there is little overlap in active and abnormal brain regions outside of the basal ganglia (Figure 3, Figure 4, Figure 5, Figure 6, Figure 7 and Figure 8). An exception to this is found in the ACC and temporal lobe with regards to auditory and temporal perception, which perhaps relates to the relationship between internal timekeeping and internal “counting”. These findings highlight the importance of the basal ganglia in sensory perception, which has not been well recognised historically.

The repeated demonstration of a multisensory response within the basal ganglia, along with their activation in response to tactile, visual and auditory stimuli, has clarified that while the basal ganglia may respond most strongly to proprioception, there is a clear response to the tactile, visual and auditory sensory modalities as well. Additionally, the larger magnitude of the neuronal response within the basal ganglia to multiple simultaneous modalities of sensory stimulation compared to a single modality [20] suggests that the role of the basal ganglia in sensory perception may not be only as a node in a relay network, but as a processing facility for integrating various simultaneous sensory inputs.

### 4.2. What Could Be the Reason for Reduced Sensory Function in Parkinson’s Disease?

Increased noise or decreased specificity in the PD sensory system [36,71,72,73,74,75,76,77,78] may be attributed to various factors including an inherent reduction in the specificity of basal ganglia signalling in response to sensory stimuli [36,71,75], an increase in receptive field size considering the somatopic organization of the basal ganglia sensory system [53,56,58,59,60,61,62,63,67,68,69,79] and potentially continuous proprioceptive signalling, due to impaired muscular relaxation in PD, leading to overburdening of neuronal firing or noise [80]. Indeed, this loss of specificity may extend beyond the basal ganglia as Escola [72] found evidence of decreased cortical specificity to sensory responses via MER in the pre-SMA and SMA in primate models of PD. Somatosensory-evoked potential studies in PD cohorts have also revealed a number of features such as enlarged high-frequency oscillations in the S1 region [81,82], which are possibly caused by the high burden of proprioceptive signalling. The hypothesis that reduced sensory response in PD may be related to increased proprioceptive noise caused by high resting muscular tone is supported by the Pierantozzi [83] study which revealed that a reduced somatosensory-evoked potential N30 response in PD had a remarkable increase in amplitude after a peripheral nerve block that reduced the tone. The Onofrj [84] study further supported this idea by showing that the equivalent somatosensory-evoked potential response in animals improves after a GABA (γ-Aminobutyric acid) activating anaesthetic agent, as opposed to dopaminergic medication, possibly due to reduction in tone by the former.

### 4.3. How Does the Brain Integrate Sensory and Motor Function, and What Is the Significance of This with Regard to Parkinson’s Disease?

‘Sensorimotor gating’ observed in non-PD populations shows that motor neuronal activity can inhibit sensory neuronal activity [14,50,80,85,86] and sensory neuronal activity can inhibit motor neuronal activity [87,88]. This phenomenon is reduced in PD [14,15,80,87,88,89], potentially impacting the precision of motor commands [88] and possibly contributing to excess motor output. Conversely, a high burden of proprioceptive noise is likely to lead to reduced motor output in PD due to sensorimotor gating [16]. These irregularities in the normal homeostatic mechanisms of motor control in PD may underlie the paradox of excessive motor output (increased tone) and reduced motor output (bradykinesia).

## 5. Methodological Limitations within the Reviewed Studies

### The Recurrent Problem of Determining Brain Response Related to a Pure Sensory Stimulus in the Setting of Confounds

Several studies emphasize the importance of the contralateral S1 in the somatosensory temporal-discrimination task [22,49,50,85,86,90,91,92,93,94,95] but there was no clear distinction between temporal and tactile processing. This ambiguity extends to other brain areas as well, with studies such as Pastor [26] and Lacruz [22] reporting involvement of the caudate in temporal processing but not controlling for the confound of brain-area activation to other forms of tactile stimuli. Additionally, Ferrandez [23] and Schmiedt [41] have highlighted examples of frontal-brain activity in sensory perception, suggesting higher-order “top-down” sensory processing occurring concurrently or shortly after pure sensory perception. The hypothesis is that these higher-order areas are distinct from brain regions involved in pure sensory perception and reflect processes of categorization, classification, problem solving, and working memory. These outcomes imply that the most robust evidence for brain-area involvement in pure sensory perception will be derived from studies that isolate brain areas activated by utilizing varying experimental paradigms to interrogate a single sensation perception.

## 6. Limitations and Future Directions

### 6.1. Limitations within the Present Review

Despite extensive efforts to encompass a vast array of studies probing brain networks involved in sensory function and abnormalities in PD, some relevant studies, especially non-English ones, might have been overlooked. However, multiple reviewers screened the papers and there was no date restriction for publication, with the search strategy including all databases. The reviewing team included clinicians and a neuroscientist for a broader interpretation.

There are established connections between ascending sensory pathways and the circuits involved in novelty-reward processing (including the nucleus accumbens). However, studies with experimental paradigms designed to assess the novelty-reward dopaminergic system in combination with sensory testing were not captured within our inclusion/exclusion criteria (see the Supplementary Materials). The question of involvement of the novelty-reward dopaminergic system therefore could not be addressed within this review. However, it is conceivable that if this system was activated by the sensory-testing experimental paradigm, PwPD would have a reduced response due to the reduction in dopaminergic function in this area. This is an important consideration that should be addressed in a future literature synthesis. Notably, the sensory-testing methodologies that were included in our review (see Section 3.2.2) included no rewards for participation in activities, nor for achieving a goal performed through sensory system activation, for animals or humans. Animal studies in particular usually occurred in a restrained animal which was exposed to a pure sensory stimulus. It is conceivable that the motivation-reward circuit may have activated in humans who wanted to do well in a task; however, there was generally no feedback for participants as to whether responses were good/bad or correct/incorrect. As such, any activation of reward circuitry would likely be intrinsic to the tasks (i.e., stimulus novelty) rather than explicit to the experimental designs.

### 6.2. Difficulty Interrogating Deep Brain Nuclei for Sensory Function in Human Models

Inconsistencies emerged in studies focusing on individual sensory systems, particularly those interrogating the basal ganglia, due to challenges in controlling for co-stimulation or cross-talk, and the limitations of existing technologies like fMRI, PET, and surface EEG in studying small deep nuclei. Additionally, human MER is not possible in the research setting unless in the context of a therapeutic intervention, which therefore limits the studied population to diseased cohorts. A potential technology that may be able to provide further insights is brain-focused ultrasound, which has the ability to reversibly modulate the function of deep brain nuclei in a non-invasive procedure, with a neuromodulation focus as small as 2 mm [96]. Another potential method to interrogate sensory function of deep nuclei would be to test various sensory functions before and after DBS implantation [8].

## 7. Conclusions

This review described brain-area activation in HC and abnormalities in PD associated with tactile, proprioceptive, auditory, visual, and temporal sensory modalities. In PD, the basal ganglia are consistently implicated in sensory dysfunction across all sensory modalities. The sensory dysfunction may be attributed to concepts like “increased noise” and “decreased neuronal specificity,” possibly due to factors like high resting muscular tone, increased receptive field size of sensory neurons, and inherent decreased specificity at the basal ganglia. Sensory-system dysfunction may have significant clinical importance as it may, in part, lead to motor-system dysfunction due to sensorimotor gating. Irregularities in the normal homeostatic sensorimotor mechanisms of motor control in PD may underlie the paradox of excessive motor output (increased tone) and reduced motor output (bradykinesia). Irrespective of their impact on motor function, sensory abnormalities are also associated with reduced function and quality of life in their own right (Chaudhuri and Schapira 2009). The analysis of sensory-system dysfunction therefore may be paramount in understanding functional disability in PD.

Exploring brain networks involved in sensory modalities in normal populations and assessing the associated dysfunctions in PD offers valuable insights into the pathological processes in PD and their functional implications. Despite the historical focus on the motor aspects of PD, there is a growing realization that PD is more than just a motor disorder. In PD, the abnormalities in the sensory system have, until now, been relatively underexplored. Only through increased focus on the sensory abnormalities can we hope to disentangle this component of the disease and hence start to find new strategies to treat the whole of PD.

## Data Availability

The extracted data from this review is available in the Supplementary Materials.

## Supplementary Materials

The following supporting information can be downloaded at: https://www.mdpi.com/article/10.3390/brainsci13111552/s1. Refs. [97,98,99,100,101,102,103,104,105,106,107,108,109,110,111,112,113,114,115,116,117,118,119,120,121] are cited in the Supplementary Materials.

## Abbreviations

ACC	anterior cingulate cortex
DBS	deep brain stimulation
DLPFC	dorsolateral prefrontal cortex
EEG	electroencephalogram
fMRI	functional magnetic resonance imaging
GABA	γ-Aminobutyric acid
GPe	globus pallidus externa
GPi	globus pallidus interna
HC	healthy control
IPL	inferior parietal lobule
MER	microelectrode recording
MMN	mismatch negativity
MPTP	1-methyl-4-phenyl-propionoxy-piperidine
PD	Parkinson’s disease
PET	positron emission tomography
PPC	posterior parietal cortex
PwPD	people with Parkinson’s disease
SMA	supplementary motor area
SN	substantia nigra
STN	subthalamic nucleus
TMS	transcranial magnetic stimulation
