# Supplementary material for: Brain Networks Involved in Sensory Perception in Parkinson’s Disease: A Scoping Review
Source: Brain Sci. 2023 Nov 6;13(11):1552. doi: 10.3390/brainsci13111552 (PMC10669548; doi:10.3390/brainsci13111552)
Supplement: Supplementary file 1 [file brainsci-13-01552-s001.zip › brainsci-2687452_ Supplementary.pdf]

Contents

S1. Supplementary Methods Information

1. Inclusion Criteria
2. Exclusion Criteria
3. Systematic Search Strategy

S2. Extracted Data

1. Sample Characteristics
2. Data extracted from the search strategy for the brain areas active during sensory activation in healthy and Parkinsonian models.
3. Data extracted from the search strategy for the brain areas active during the mixed proprioceptive and tactile SEP response in healthy and Parkinsonian models.
4. Somatopic organisation of nuclei in the basal ganglia.
5. Studies demonstrating sensorimotor integration.
6. Studies demonstrating increased noise / decreased specificity at the basal ganglia in Parkinson's disease

Abbreviations

Cohorts: HC = healthy cohort, PD = Parkinson's disease, PSP = progressive supranuclear palsy, HD = Huntington's disease.

Brain areas: S1 = primary somatosensory cortex, DLPFC = dorsolateral prefrontal cortex, pre-SMA = pre-supplementary motor area, SPC = superior parietal cortex, PPC = posterior parietal cortex, STG = superior temporal gyrus, ACC = anterior cingulate cortex, SMA = supplementary motor area, VL PFC = ventrolateral prefrontal cortex, PPC = posterior parietal cortex, MT/V5 = visual motor area, STN = subthalamic nucleus, VL thalamus = ventrolateral thalamus, GPe = globus pallidus externa, GPi = globus pallidus interna, GP = globus pallidus, ENTO = entopeduncular nucleus, STAC = superior temporal auditory cortex, IPC = inferior parietal cortex, S2 = secondary somatosensory cortex, M1 = primary motor cortex, PPN = pedunculopontine nucleus.

Sensory tests: STDT = somatosensory temporal discrimination threshhold, Third DT = third somatosensory temporal discrimination threshhold, TOJ = temporal order judgement,

Brain area involvement interrogation methods: EEG = electroencephalogram, PET = positron emission tomography, fMRI = functional magnetic resonance imaging, TMS = transcranial magnetic stimulation, rTMS = repetitive transcranial magnetic stimulation, ppTMS = paired pulse transcranial magnetic stimulation, cTBS = continuous theta band stimulation, SEP = somatosensory evoked potential, tACS = transcranial alternating current stimulation, MEG = magnetoencephalogram, AEP = auditory evoked potential, N1 = first vertex-negative deflection auditory evoked response, P3 = third vertex-positive deflection in auditory evoked response, MMN = mismatch negativity, ERP = event related potential, VEP = visual evoked potential, P100 = vertex-positive deflection at 100msec on visual potential, PERG = pattern electroretinogram, P50 = vertex-positive deflection at 50msec in pattern electroretinogram.

Disease severity markers: H&Y = Hoehn & Yahr score ("OFF meds" or not stated), UIII = UPDRSIII score ("OFF meds" or not stated), LEDD = levodopa effective daiy dose, MMSE = mini mental state examination.

Other: c/l = contralateral, i/l = ipsilateral, hf = high frequency, b/l = bilateral, L = left, R = right, DT = discrimination threshhold, DD = disease duration, DBS = deep brain stimulation, MPTP = 1-methyl-4-phenyl-1,2,3,6-tetrahydropyridine, Ldopa =levodopa, Rx = treatment.

S1 – Supplementary Methods information

1. Inclusion Criteria

The full inclusion criteria are as follows: Included sensory modalities are visual, auditory, tactile, proprioceptive and temporal. The retina is considered part of the brain for the purposes of these criteria. Publications assessing brain activity in "normals" during "pure" sensory testing. Publications assessing brain activity in Parkinson's patients during "pure" sensory testing. Publications assessing brain activity in animals during "pure" sensory stimulation. Publications assessing brain areas involved in multisensory and sensorimotor integration in the Basal Ganglia and in Parkinson's disease. Publications assessing Event Related Potentials in Parkinson's patients (including SEP, AEP and VEP). Publications investigating somatopic organization of nuclei in the Basal Ganglia. Publications demonstrating the increased "noise" / decreased specificity in the Basal Ganglia in Parkinson's disease.

2. Exclusion Criteria

The full exclusion criteria are as follows: Publications regarding non-Parkinsonian pathology (e.g dystonia, MS). Publications exploring the effects of PD dementia/MCI, unless a PD cognitively normal cohort are also assessed against "normals". Publications regarding pain, olfaction or vestibular sensation as the sensory modality. Review publications. Publications assessing whole brain resting state connectivity in Parkinsons. Publications addressing the ideal site of DBS that do not involve assessment of basal ganglia response to passive movement of joints. Publications utilizing Positron Emission Tomography (PET) to differentiate Parkinsonian conditions. Publications addressing sensory discrimination or proprioception abnormalities in PD without interrogation of brain networks involved. Publications with sensory testing also involving the dopaminergic-reward system.

3. Systematic Search Strategy

1	"parkinson*".m_titl.	11	5 and 10	21	somatosensory evoked potential*.mp.	31	neuronal response*.mp.	41	multisensory.mp.	51	positron emission tomography.m_titl.
2	somatosensory evoked potential*.mp.	12	microelectrode.mp.	22	20 and 21	32	neuronal mechanism*.mp.	42	multisensory integration.mp.	52	46 or 47 or 48 or 49 or 50 or 51
3	1 and 2	13	deep brain stimulation.mp.	23	"parkinson*".m_titl.	33	31 or 32	43	sensorimotor integration.mp.	53	44 and 52
4	subthalamic.m_titl.	14	12 and 13	24	basal ganglia.m_titl.	34	propriocep*.mp.	44	34 or 35 or 36 or 37 or 38 or 39 or 40 or 41 or 42 or 43	54	27 and 30
5	"parkinson*".m_titl.	15	6 or 7 or 14	25	striatum.m_titl.	35	kin?esthe*.mp.	45	33 and 44	55	27 and 45
6	firing rate.mp.	16	11 and 15	26	"globus pallid*".m_titl.	36	sensorimotor.mp.	46	functional brain.m_titl.	56	27 and 53
7	neural noise.mp.	17	temporal discrimination.mp.	27	23 or 24 or 25 or 26	37	visual.mp.	47	PET.m_titl.	57	54 or 55 or 56
8	basal ganglia.m_titl.	18	somatosensory.mp.	28	passive movement*.mp.	38	auditory.mp.	48	fmri.m_titl.	58	3 or 16 or 19 or 57
9	"globus pallid*".m_titl.	19	17 and 18	29	passive joint*.mp.	39	tactile.mp.	49	functional MRI.m_titl.	59	"parkinson*".m_titl.
10	4 or 8 or 9	20	"parkinson*".m_titl.	30	28 or 29	40	somatosensory.mp.	50	functional magnetic.m_titl.	60	PINK*.mp.

61	parkinson.mp.	71	visual.m_titl.	81	"sensation*".m_titl.	91	63 or 90	101	stimulation.mp.	111	99 or 101 or 109 or 110
62	60 and 61	72	tactile.m_titl.	82	"sensor*".m_titl.	92	"propriocep*".m_titl.	102	100 and 101	112	63 and 69 and 89
63	59 or 62	73	spatial.m_titl.	83	TOJ.mp.	93	"kin?esthe*".m_titl.	103	99 or 102	113	91 and 98
64	"discriminat*".m_titl.	74	auditory.m_titl.	84	TDT.mp.	94	haptic.m_titl.	104	interval timing.mp.	114	91 and 103 and 105
65	acuity.m_titl.	75	frequency.m_titl.	85	temporal.m_titl.	95	passive motion.m_titl.	105	83 or 84 or 86 or 87 or 92 or 93 or 97 or 104	115	63 and 89 and 103 and 106
66	perception.m_titl.	76	tone.m_titl.	86	temporal order judgement.mp.	96	postural sway.m_titl.	106	discriminat*.mp.	116	91 and 108 and 111
67	sensitivity.m_titl.	77	"somatosens*".m_titl.	87	temporal discrimination.mp.	97	time estimation task.mp.	107	postural instability.m_titl.	117	112 or 113 or 114 or 115 or 116
68	"abnormal*".m_titl.	78	contrast.m_titl.	88	time.m_titl.	98	92 or 93 or 94 or 95 or 96 or 97	108	96 or 107	118	limit 117 to english language
69	64 or 65 or 66 or 67 or 68	79	two point.m_titl.	89	70 or 71 or 72 or 73 or 74 or 75 or 76 or 77 or 78 or 79 or 80 or 81 or 82 or 83 or 84 or 85 or 86 or 87 or 88	99	DBS.mp.	109	medication.mp.	119	58 or 118
70	colo?r.m_titl.	80	two-point.m_titl.	90	parkinson*.mp.	100	STN.mp.	110	pallidotomy.mp.		

S2 - Extracted Data

1. Sample Characteristics.

Of the 89 human studies there were 22 that utilized healthy subjects only, 20 with PD subjects only and 44 with PD and healthy control (HC) subjects. There were 13 animal studies, 1 studying PD animals only, 5 studied animals before and after a PD model, 5 studying HC animals only, and 2 studying HC vs PD animals. Of the 2853 human subjects 1161 were HC and 1692 were pwPD. Of the 125 animals studied 22 of these were studied before and after PD model, 39 were PD model only and 64 of these studied as HC. The breakdown of animals across the studies was rats (n= 87), cats (n= 15) and monkeys (n=23).

Of the 13 studies assessing the basal ganglia for somatotopic organization [1–12], 11 utilized a PD human cohort, with a total of 754 PD subjects and 2 [4,5] assessed 4 non-PD monkeys (see S2.4).

Of the 9 studies investigating increased noise or decreased specificity in the basal ganglia in PD, Steigerwald [13], Remple [14] and Vyas [15] utilized human subjects, with a total of 91 subjects. There were a total of 11 non-PD human subjects utilized as controls in the Steigerwald and Vyas studies, with Remple [14] comparing early and late PD. Pelled [16], Mallet [17], Escola [18], Rothblat [19], Erez [20] and Pessiglione [21] utilized animal models of PD (see S2.6), with 47 PD and 39 HC animals (rats, monkeys and cats).

Of the two studies that interrogated multisensory integration in the basal ganglia [19,22], both studies utilized HC cats (n= 11) as subjects, with only Rothblat [19] also assessing cats in the PD state (n=4).

Forty-eight studies evaluated brain area activation during sensory stimulation (temporal, auditory, tactile, visual, proprioceptive) in either PD or HC or both [19,23–71]. Of these, 4 assessed

the visual evoked response [39,52,58], one assessed the auditory evoked response [63], and 1 study assessed multisensory integration [19]. Further details are outlined in S2.2.

Please see Figure 2 for further breakdown of the numbers of studies extracted between disease state, animal or human cohort and sensory modality. Figure 3 demonstrates the breakdown of brain interrogation method utilised. Further information on sample characteristics including sample size, sensory task, brain area interrogation modality and findings are presented in S2.2.

Brain interrogation modalities used to assess brain area activation during sensory testing included fMRI, MER, PET, TMS, electroencephalogram (EEG) and event related potential (ERP). fMRI, PET and TMS were only utilized in human studies, whereas ERP was utilized in one animal study, and MER provided the bulk of information in animal studies with 4 animal studies compared to 3 human studies. MER were also the predominant modality to interrogate basal ganglia activation during sensory stimulation, with the other modality utilized being fMRI. PET, TMS and surface EEG or ERP were only utilized for cortex interrogation. See Figure 3 for further details.

2. Data extracted from the search strategy for the brain areas active during sensory activation in healthy and Parkinsonian models.

Cortical networks involved in Temporal Perception from healthy cohort

Author Year	No. Subjects	Brain area involved	Other finding/comment	Task	Interrogation modality
Akatsuka 2005	8 HC		Different cortical networks involved if STDT is difficult or easy. Did not control for tactile response.	STDT	EEG
Hannula 2008	6 HC	S1 (c/l)	Did not control for tactile response.	STDT	TMS (Monophasic)
Conte 2016	17 HC	S1 (c/l)	Did not control for tactile response.	STDT	cTBS
Lei 2018	22 HC	S1 (c/l)	Did not control for tactile response.	STDT	EEG (SEP)
Rai 2012	18 HC	S1 (c/l)	Did not control for tactile response.	STDT	cTBS
Conte 2012	10 HC	S1 (c/l), *not pre-SMA (c/l), DLPFC (c/l) or cerebellum (i/l*)	Did not control for tactile response.	STDT	cTBS
Leodori 2017	31 HC	S1 (c/l)	Even more active in detecting third stimulus. Did not control for tactile response.	STDT, Third DT: Standard STDT plus a third stimulus to compare.	TMS
Rocchi 2017	15 HC	S1 (c/l)	Increased inhibition in S1 improves STDT ability. Did not control for tactile response.	STDT	TMS(hf)
Rocchi 2016	22 HC	S1 (c/l)	Increased cortical inhibition important for temporal discrimination ?due to decreased noise. Did not control for tactile response.	STDT	cTBS
Baumgarten 2015	16 HC	S1 (c/l)	Phase of alpha and slow beta in S1 at time of stimulus affect STDT ability. Did not control for tactile response.	STDT	MEG

Baumgarten 2017	25 HC	S1 (c/l)	Subliminal stimulus can reset phase in S1 and thus affect STDT. Did not control for tactile response.	STDT	MEG
Manzo 2020	17 HC	*not S1 (c/l) (?tACS unable to activate deep S1 layers)*		STDT	tACS
Lacruz 1991	84 HC, 51 with focal lesions	S1, PPC, SMA	Did not control for tactile response.	STDT	Lesion study
Otsuru 2019	36 HC	PPC (b/l if left PPC, c/l if right PPC), *not S1*	Left PPC stimulation, in additional to contralateral affect, has some ipsilateral effect on TOJ. Did not control for tactile response.	TOJ (L + R): Electrical pulses to ring electrode on digit, patient determines order of stimuli.	tACS
Pastor 2004	14 HC	pre-SMA (b/l) and anterior cingulate (b/l) (specific to temporal), *not SPC (R) and precuneus (R)*	R postcentral gyrus, inferior parietal lobule, middle and inferior frontal gyri, R insula in anterior aspect and R anterior cingulate (both temporal and spatial discrimination)	STDT	fMRI
Bolognini 2010	13 HC	S1 (c/l) and STG (c/l)	TMS over S1 also affects tactile spatial function, whereas TMS over STG does not.	STDT	TMS
Huang 2022	40 HC	ACC (b/l) and pre-SMA (c/l), medial temporal lobule (i/l), SMA (i/l) present (temporal vs spatial)	Connection to each other and dorsal putamen important	STDT	fMRI
Pastor 2006	14 HC	pre-SMA (R) and anterior cingulate (R) (temporal vs spatial)		Temporal Auditory DT: Binaural paired clicks with differing intensities b/w ears.	fMRI
Ferrandez 2002	11 HC	SMA (b/l), VL PFC (b/l), premotor (L), inferior parietal (b/l), temporal gyri (b/l) (temporal vs intensity).		Visual Duration Discrimination (b/l stimulus) : Duration and intensity of LED light compared between current and previous.	fMRI
Bueti 2008	10 HC	PPC (R) (visual temporal and auditory temporal) and MT/V5		Visual and Auditory Duration Discrimination (b/l stimuli): Duration discrimination	rTMS

		(visual temporal only) (L (but hypothesized to also be active on R))		between two visual stimuli with 1moving stimuli and 2) static stimuli. 3) Visual pattern recognition. 4) Auditory duration discrimination.	
Elsinger 2003	10 PD, 13 HC	STG (b/l) (auditory temporal and memory temporal), SMA (b/l) (memory temporal / internal timekeeping only).	STG activation reduced in memory condition compared to auditory condition but still present ?merely due to "internal voice". Sensorimotor cortex and cerebellar activation (auditory temporal and memory temporal) hypothesized to be due to the finger tapping required in both conditions.	Time (pace) estimation: Paced tone binaurally, with participant matching pace with finger taps, then auditory stimulus stopped and participant asked to continue pace.	fMRI

Cortical networks involved in Temporal Perception from Parkinson's patients

**Author Year**	**No. Subjects**	**Disease Severity**	**Brain area involved**	**Other finding/comment**	**Task**	**Interrogation modality**
Conte 2010	13 PD, 13 HC	DD 7-20 years	S1 (measured centrally)	S1 activity (parietal component of SEP) decreased with DBS-on, which also worsened STDT regardless of side stimulated (but only when patients also on Ldopa). Did not control for tactile response.	STDT	EEG (SEP)
Koch 2004	10 PD, 10 HC	DD 4.6 ± 1.8 years. H&Y<2. UIII 35±4. LEDD 400 ± 125mg	Temporal ability improved after DLPFC (R) TMS *(but not SMA (b/l))* in PD, but not for HC	Did not control for affect of TMS on visual ability.	Visual Time Reproduction Task: Visual stimulus duration replication (with button press).	rTMS
Dusek 2012	12 PD	DD 6.9 ± 3.2 years	Precuneus (b/l)	Precuneus more active in PD patients ON medication (who performed better) in the time reproduction phase than PD patients OFF medication. Noted both in the encoding (visual temporal) (approaching	Visual Time Reproduction Task: Visual stimulus duration replication (with button press).	fMRI

				significance) and continuation (memory temporal) (significant) conditions.		
Elsinger 2003	10 PD, 13 HC	DD 3.9 (3-7) years.<br>H&Y 1-2	SMA (b/l) active during continuation condition (memory temporal / internal timekeeping only).	Active only in PD patients ON medication and HC, reduced in PD OFF medication. ?SMA activity due to pure temporal effect vs. memory retrieval for continuation condition.	Time (pace) estimation: Paced tone binaurally, with participant matching pace with finger taps, then auditory stimulus stopped and participant asked to continue pace.	fMRI

Basal Ganglia/Cerebellar networks involved in Temporal Perception from healthy cohort

Author Year	No. Subjects	Brain area involved	Other finding/comment	Task	Interrogation modality
Lacruz 1991	84 HC, 51 with focal lesions	Basal ganglia (putamen, caudate) & mediolateral thalamus	Did not control for tactile response.	STDT	lesion study
Huang 2022	40 HC	Dorsal putamen (c/l) (temporal vs spatial)	Dorsal putamen connection to ACC and pre-SMA important	STDT	fMRI
Nenadic 2003	15 HC	Putamen (R) (temporal vs frequency).		Auditory temporal discrimination threshold: Tone pairs binaurally that different in pitch.	fMRI
Ferrandez 2003	11 HC	Putamen (L) (temporal vs intensity).		Visual Duration Discrimination (b/l stimulus) : Duration and intensity of LED light compared between current and previous.	fMRI
Pastor 2004	14 HC	Head of Caudate, STN, Substantia Nigra, Cerebellar Vermis and Crus II (all B/L) (& Thalamus) (temporal vs spatial)	Active in both temporal and spatial discrimination	STDT	fMRI

Basal Ganglia/Cerebellar networks involved in Temporal Perception from Parkinson's patients

Author Year	No. Subjects	Disease Severity	Brain area involved/abnormal	Other finding/comment	Task	Interrogation modality
Di Biasio 2015	15 PD, 10 HC	H&Y 1.4± 0.5<br>UIII 11.53 ± 5.9	Cerebellum (i/l)	Cerebellar cTBS stimulation improved STDT scores only for PD patients in the OFF condition, suggesting the cerebellum may act as a compensatory system in PD. Did not control for brain activity involved in tactile processing.	STDT	cTBS
Elsinger 2003	10 PD, 13 HC	DD 3.9 (3-7) years.<br>H&Y 1-2	Putamen (c/l), VL thalamus (c/l) during continuation condition (memory temporal / internal timekeeping) only.	Putamen and thalamus only active in PD patients ON, but not OFF medication. ?activity due to pure temporal effect vs. memory retrieval for continuation condition.	Time (pace) estimation: Paced tone binaurally, with participant matching pace with finger taps, then auditory stimulus stopped and participant asked to continue pace.	fMRI

Cortical networks involved in Auditory Perception from healthy cohort

Author Year	No. Subjects	Brain area involved	Other finding/comment	Task	Interrogation modality
Pastor 2006	14 HC	Middle and inferior frontal gyri (R), anterior cingulate (R), insula (R) (both auditory temporal and auditory spatial)		Temporal Auditory DT: Binaural paired clicks with differing intensities b/w ears.	fMRI
Nenadic 2003	15 HC	STG (b/l), medial PFC (R) and DLPFCs (b/l), anterior		Auditory temporal discrimination threshold: Tone pairs binaurally that different in pitch.	fMRI

		cingulate (L) activation (both duration and frequency)			
Elsinger 2003	10 PD, 13 HC	STG (b/l) (auditory pace > memory pace)		Time (pace) estimation: Paced tone binaurally, with participant matching pace with finger taps, then auditory stimulus stopped and participant asked to continue pace.	fMRI

Basal Ganglia networks involved in Auditory Perception from healthy cohort

Author Year	No. Subjects	Brain area involved	Other finding/comment	Task	Interrogation modality
Pastor 2006	14 HC	Head of caudate and putamen (R)		Temporal Auditory DT: Binaural paired clicks with differing intensities b/w ears.	fMRI

Basal Ganglia networks involved in Auditory Perception from "healthy" animals

Author Year	No. Subjects	Brain area involved	Other finding/comment.	Task	Interrogation modality
Nagy 2006	7 Cats	Caudate: 6% of neurons responded to auditory stimuli. Substantia Nigra also revealed a response to auditory stimuli.		White noise at 60 db for 1 second.	Microelectrode recording
Rothblat 1995	4 Cats	GPe (GP) 22% responded to auditory stimulation	Cats tested before MPTP model for Parkinsonism.	Multifrequency click (5 ms duration, 1s frequency) were	Microelectrode recording

		GPi (ENTO) 8% responded to auditory stimulation		delivered by means of a centrally located loudspeaker	

Basal Ganglia networks involved in Auditory Perception from Parkinsonian animal models

Author Year	No. Subjects	Brain area involved	Animal model	Task	Interrogation modality
Rothblat 1995	4 Cats	GPe (GP) 5% responded to auditory stimulation (reduced)<br>GPi (ENTO) 4% responded to auditory stimulation reduced)	MPTP model	Multifrequency click (5 ms duration, 1s frequency) were delivered by means of a centrally located loudspeaker	Microelectrode recording

Cortical networks involved in the Auditory Evoked Responses in Parkinson's patients

Author Year	No. Subjects	Disease Severity	PD abnormality	Changes with medication	Changes with DBS	Other/Comment	Task	Interrogation modality
Pekkonen 1995	13 PD, 11 HC	H&Y =1 (10/13 pts) H&Y =2 (3/13 pts)	STAC = N1 generation site (midline) lower amplitude in PD vs controls. Frontal and STAC = MMN generation site (midline) was lower amplitude in PD than controls.	STAC initially reflects Auditory stimulus detection. MMN reflects Auditory discrimination ability.			Auditory frequency discrimination: Deviant tone played in sequence of standard tones.	EEG (ERP)
Philipova 1997	17 PD, 17 HC	DD 2.9 mean years	STAC = N1 generation site (midline) and Frontal = P3 generation site (midline) lower amplitude in PD vs controls.	STAC initially reflects Auditory stimulus detection. Frontal P3 reflects stimulus evaluation and discrimination			Auditory frequency discrimination: high and low tones in sequence. After each tone button press for high vs low.	EEG (ERP)
Rossi 1985	5 PD, 20 HC	Early PD before	Reduced amplitude (one or more peaks absent) in 3/5 PD, prolonged	Return mostly to normal with 2 months of Ldopa treatment		No full text available	Auditory clicks presented monaurally	EEG (AEP)

		initiating Ldopa Rx.	latencies (interpeak and absolute)					
Weise 2015	50 PD, 50 HC	*Not stated.*	*Auditory Evoked Potential equivalent latency (amplitude not measured) between PD and HC*				Auditory click stimulus	EEG (AEP)

Cortical networks involved in Visual Perception from healthy cohort

Author Year	No. Subjects	Brain area involved	Other finding/comment	Task	Interrogation modality
Ferrandez 2003	11 HC	Superior occipital gyrus (R), fusiform gyri (b/l), hippocampus (L), precuneus (L), posterior intraparietal sulcus (L), prefrontal cortex (L), thalamus (R pulvinar, mediodorsal nucleus, bilateral geniculate nuceli) active in Intensity but not Duration discrimination		Visual Intensity Discrimination (b/l stimulus) : Duration and intensity of LED light compared between current and previous.	fMRI
Bueti 2008	10 HC	*No effect of rTMS on pattern recognition when applied over vertex, right IPC or left V5/MT*		Visual and Auditory Duration Discrimination (b/l stimuli): Duration discrimination between two visual stimuli with 1moving stimuli and 2) static stimuli. 3) Visual pattern recognition. 4) Auditory duration discrimination.	rTMS
Schmiedt 2005	14 PD, 10 HC	Occipital (b/l), Frontal (L)	Early occipital excitability = low-level visual processing. Early left frontal	Visual Shape Recognition (b/l)	EEG (ERP)

			synchronization may reflect top-down processing including categorization and classification.		

Cortical networks involved in Visual Perception from Parkinson's patients

Author Year	No. Subjects	Disease Severity	Brain area involved	Other finding/comment	Task	Interrogation modality
Schmiedt 2005	14 PD, 10 HC	DD 3.6 ± 3.4 years. UIII 18 ± 12.6 LEDD 257 ± 59mg.	Increased early posterior (R) activity, and decreased frontal (L) activity in PD (but not controls).	Early posterior (R) activity reflects strategy of attention to pattern rather than category. Decreased frontal (L) activity reflects reduced top-down processing including categorization and classification	Visual shape discrimination: Eight shapes presented as targets or distracter. Button press when target presented.	EEG (ERP)
Cardoso 2010	16 PD, 18 HC	DD 10 ± 4.3 years. UIII 32.8 ± 8.74. H&Y 2.25 ± 0.32. LEDD 1009mg avg.	V1 (b/l) reduced with flickering checkerboard, increasde fusiform gyrus (R) with emotional facial discrimination.		1) Stare at flickering checkerboard, 2) Define facial emotion (mild vs sig. sadness), 3) Facial gender discrimination.	fMRI

Basal Ganglia networks involved in Visual Perception from “healthy” animals

Author Year	No. Subjects	Brain area involved	Other/comment	Task	Interrogation modality
Nagy 2006	7 Cats	Caudate: 17% of neurons responded to visual stimuli. Substantia Nigra also revealed a response to visual stimuli.		Light spots for 1 second with optimum moving direction and velocity for each neuronal unit.	Microelectrode recording
Rothblat 1995	4 Cats	GPe (GP) 16% responded to visual stimulation<br>GPi (ENTO) 11% responded to visual stimulation	Cats tested before MPTP model for Parkinsonism.	Red light from emitting diodes 250cm in front of cat for 5ms.	Microelectrode recording

Basal Ganglia networks involved in Visual Perception from Parkinsonian animal models

Author Year	No. Subjects	Brain area involved	Animal model	Task	Interrogation modality
Rothblat 1995	4 Cats	GPe (GP) 1 % responded to visual stimulation (reduced)<br>GPi (ENTO) NO response to visual stimulation.	MPTP model	Red light from emitting diodes 250cm in front of cat for 5ms.	Microelectrode recording

Cortical networks involved in the Visual Evoked Response in Parkinson's patients

Author Year	No. Subjects	Disease Severity	PD VEP abnormality	Changes with medication	Changes with DBS	Other/Comment	Task	Interrogation modality
Okuda 1996	18 nD-PD, 11 D-PD, 11 Bins-wanger's, 11 HC	H&Y > 3 (10/11 D-PD).<br>H&Y > 3 (11/18 nD-PD).	Only PD with dementia had prolonged P100 c/w controls.	Ldopa prolongs P100 latency		Prolongation of P100 correlated with age, illness duration and MMSE score, but not H&Y score	Stare at black and white checkerboard on screen, monocular, pattern reversing at 1Hz	EEG (VEP)
Kupersmith 1982	28 PD, 28 HC	H&Y 1-5.	Increased latency in P100 for PD with H&Y>1			P100 latency increased with increasing H&Y stage	Stare at vertical grating with 2cps cycling and 3 cycles per degree spacing.	EEG (VEP)

Basal Ganglia networks involved in the Visual Evoked Response in Parkinson's patients

Author Year	No. Subjects	Disease Severity	PD VEP abnormality	Changes with medication	Changes with DBS	Other/Comment	Task	Interrogation modality
Pesenti 2003	8 PD	UIII 41.7 ± 10.1. LEDD 1290 ± 495mg	*Failed to detect components at P100 latency at STN, or after visual stimulation.*				not defined	Microelectrode at STN (VEP)

Cortical networks involved in the Visual Evoked Response in Parkinsonian animal models

Author Year	No. Subjects	PD VEP abnormality	Changes with medication	Changes with DBS	Animal model	Task	Interrogation modality
Ghilardi 1998	5 Cynomolgus monkeys	Prolonged latency and reduced amplitude in PD monkeys, which partially returned to normal as PD symptoms resolved			MPTP model	Stare at vertical grating with spatial frequencies 0.5, 1.2, 2.5 and 3.5 cycles/deg and with counterphase modulation at 1Hz (and for 2 monkeys at 4,6 and 8Hz)	EEG (VEP)

Visual abnormality at the retina in Parkinson's patients

Author Year	No. Subjects	Disease Severity	PD ERG abnormality	Changes with medication	Changes with DBS	Other/Comment	Task	Interrogation modality
Kupersmith 1982	28 PD, 28 HC	H&Y 1-5.	*ERG failed to differentiate PD from HC*			ERG potentials are expected to reflect the inner nuclear layer of the retinal neuronal activity - cells which contain dopamine	Stare at a pair of bright light flashes presented at 1 minute intervals. ERG at second flash.	ERG
Peppe 1992	10 PD	DD 2.3 (0.5-5) years. H&Y 2-3.	PERG P50 latency increased more with decreasing contrast in PD than HC	Ldopa normalized this effect for PD		Suggests retinal involvement of contrast insensitivity in PD which is Ldopa responsive	Stare at reversing vertical square-wave grating pattern, 2 cycles per degree, on a television screen of varying contrasts, 1m from eye.	PERG

Visual abnormality at the retina in Parkinsonian animal models

Author Year	No. Subjects	PD ERG abnormality	Changes with medication	Changes with DBS	Animal model	Task	Interrogation modality
Ghilardi 1998	5 Cynomolgus monkeys	Reduced amplitude, and delayed latency (to a smaller degree) in PD monkeys, which partially returned to normal as PD symptoms resolved			MPTP model	Stare at vertical grating with spatial frequencies 0.5, 1.2, 2.5 and 3.5 cycles/deg and with counterphase modulation at 1Hz (and for 2 monkeys at 4,6 and 8Hz)	PERG

Cortical networks involved in Tactile Perception from healthy cohort

Author Year	No. Subjects	Brain area involved	Other finding/comment	Task	Interrogation modality
Rocchi 2017	5 HC	S1 (c/l)	-	Tactile determination of grating orientation (horizontal or vertical), R&L with fingertip assessed.	TMS (hf)
Rai 2012	18 HC	S1 (c/l)	-	Tactile amplitude DT (R): Two vibratory stimulus to hand dorsum, asked to determine which had higher intensity.	TMS
Pastor 2004	14 HC	Superior Parietal Cortex (c/l) and precuneus (c/l) (spatial but not temporal), *not pre-SMA or anterior cingulate*	R postcentral gyrus, inferior parietal lobule, middle and inferior frontal gyri, R insula in anterior aspect and R anterior cingulate (both temporal and spatial discrimination)	Spatial acuity: Asked if paired electrical pulses was on R or L side of LEFT forearm	fMRI
Bolognini 2010	13 HC	S1 (c/l), *not STG* (c/l)	-	Spatial extent (long or short): Vibration stimulus to RIGHT index finger.	TMS
Huang 2022	40 HC	S1 (area 3b) (c/l), Inferior and superior parietal lobules (c/l), precuneus (c/l), visual cortex (c/l) present in Spatial NOT Temporal	-	Tactile spatial discrimination: Vibration stimulus to site on L leg, asked to judge location.	fMRI
Palomar 2011	14 PD, 13 HC	S1 (c/l)	-	Tactile amplitude DT: Ring electrode to L thumb stimulus of varying intensities. Asked to determined if stimulus detected or not.	ppTMS
Zhao 2014	21 PD, 22 HC	S1 (b/l) and middle temporal gyrus (V5), IPC (c/l), occipital (i/l), cerebellum	-	1) Two round wooden wheels: one smooth, other serrated, rolled over R index fingerpad only, 2) Active finger tapping only, 3) Sensory stimulus from (1) and asked to tap finger if detected smooth, but not serrated, wheel.	fMRI

Basal Ganglia networks involved in Tactile Perception from healthy cohort

Author Year	No. Subjects	Brain area involved	Other finding/comment	Task	Interrogation modality
Pastor 2004	14 HC	Head of Caudate, STN, Substantia Nigra, Cerebellar Vermis and Crus II (all B/L) (& Thalamus)	Active in both temporal and spatial discrimination	Spatial acuity: Asked if paired electrical pulses was on R or L side of LEFT forearm	fMRI

Cortical networks involved tactile stimulation in “healthy” animals

Author Year	No. Subjects	Brain area involved	Other finding/comment	Task	Animal	Interrogation modality
Mowery 2011	23 rats	S1(c/l)	Responsiveness declined and latencies prolonged with rate of stimulation increase	Computer controlled whisker deflection	Rats.	Microelectrode recording

Basal Ganglia networks involved tactile stimulation in "healthy" animals

Author Year	No. Subjects	Brain area involved	Other finding/comment	Task	Animal	Interrogation modality
Mowery 2011	21 rats	Dorsolateral Striatum (c/l)	Responsiveness did not decline and latencies did not prolong with increased rate of stimulation.	Computer controlled whisker deflection	Rats.	Microelectrode recordings
DeLong 1985	3 monkeys	Globus Pallidus and STN examined. Cells responding to active movement checked for response to sensation. Only GPe cells (4%) responded to muscle palpation. GPe (1%) and GPi (5%) cells responded to Tendon taps.	Only a small percentage of cells responded to tactile stimulation compared to proprioceptive stimulation (GPe 37%, GPi 22%, STN 20%).	Muscle or joint palpation, tendon taps, light touch or hair stimulation.	Monkeys.	Microelectrode recordings

		No cells in GPe, GPi or STN responded to light touch. Only 2% of GPi cells responded to body hair stimulation.				
Nagy 2006	7 Cats	Caudate: 19% of neurons responded to tactile stimuli. Substantia Nigra also revealed a response to tactile stimuli.		Light tactile stimulation 1cm2 on shaved trunk for 1 second.	Cats	Microelectrode recording
Rothblat 1995	4 Cats	GPe (GP) 31.4% responded to tactile stimulation of the face<br>GPi (ENTO) 29% responded to tactile stimulation of the face	Cats tested before MPTP model of Parkinsonism.	Probe of face with wooden probe, refined with von Frey hairs on electro-mechanical stimulator.	Cats	Microelectrode recording

Cortical networks involved in Tactile Perception from Parkinson's patients

Author Year	No. Subjects	Disease Severity	Brain area involved	Other finding/comment	Task	Interrogation modality
Weder 2000	12 PD, 12 HC	DD 7.7 ± 4.1 years.	S1 & M1 (c/l), premotor area (b/l), SMA (b/l) and parietal lobule (b/l) deficient in PD vs HC during task. INCREASED cerebellar activation.	PD patients with low FDOPA uptake in caudate (severe disease) also had deficient activation in the prefrontal cortex.	Tactile shape discrimination: Cuboid object comparison for oblongness with right hand.	PET
Zhao 2014	21 PD, 22 HC	H&Y 1-2.	S1 (b/l), parietal lobes (b/l), premotor (L) and visual cortex (R) decreased activity, increased frontal lobes (b/l) of PD patients during task c/w HC	-	1) Two round wooden wheels: one smooth, other serrated, rolled over R index fingerpad only, 2) Active finger tapping only, 3) Sensory stimulus from (1)	fMRI

					and asked to tap finger if detected smooth, but not serrated, wheel.	
Palomar 2011	14 PD, 13 HC	UIII 23 (9-33). H&Y 1-2.	*not S1(c/l)*	*ppTMS over right S1 able to improve performance in controls but not PD ON MEDS*	Tactile amplitude DT: Ring electrode to L thumb stimulus of varying intensities. Asked to determined if stimulus detected or not.	ppTMS

Basal Ganglia networks involved in Tactile Perception from Parkinson's patients

**Author Year**	**No. Subjects**	**Disease Severity**	**Brain area involved**	**Other finding/comment**	**Task**	**Interrogation modality**
Weder 2000	12 PD, 12 HC	DD 7.7 ± 4.1 years.	Cerebellum: Dentate (L), Lobulus Semilunaris (R)		Tactile shape discrimination: Cuboid object comparison for oblongness with right hand.	PET

Basal Ganglia networks involved tactile stimulation in Parkinsonian animal models

**Author Year**	**No. Subjects**	**Brain area involved**	**Animal model**	**Task**	**Interrogation modality**
Rothblat 1995	4 Cats	GPe (GP) 12.2% responded to tactile stimulation of the face (reduced)<br>GPi (ENTO) 13% responded to tactile stimulation of the face (reduced)	MPTP model	Probe of face with wooden probe, refined with von Frey hairs on electro-mechanical stimulator.	Microelectrode recording

Cortical networks involved in Proprioception from healthy cohort

Author Year	No. Subjects	Brain area involved	Other finding/comment	Task	Interrogation modality
Kalmar 2011	10 PD, 5 HC	S1(c/l), M1 (c/l) and SMA (c/l)		Passive flexion-extension of the fingers	fMRI
Boecker 1999	8 PD, 8 HD, 8 HC	S1, S2 (c/l)		High freq. vibration stimulus to immobilized metacarpal joint of the index finger	PET

Basal Ganglia networks involved in Proprioception from healthy cohort

Author Year	No. Subjects	Brain area involved	Other finding/comment	Task	Interrogation modality
Kalmar 2011	10 PD, 5 HC	Putamen (?c/l unclear) activation at times (not always)		Passive flexion-extension of the fingers	fMRI
Boecker 1999	8 PD, 8 HD, 8 HC	Globus pallidus (c/l)		High freq. vibration stimulus to immobilized metacarpal joint of the index finger	PET

Cortical networks involved in Proprioception from Parkinson's patients

Author Year	No. Subjects	Disease Severity	Brain area involved	Other finding/comment	Task	Interrogation modality
Kalmar 2011	10 PD, 5 HC	DD 6.5 (2-13) years.<br>H&Y 1.65 (1-3).	S1 (i/l), M1 (i/l) and SMA (i/l) activation increases c/w HC who have only c/l activation	• Results suggesting adaptive reorganization to less affected hemisphere.<br>• Reorganization more pronounced in R-sided dominant PD ?preserving	Passive flexion-extension of the fingers	fMRI

				function of dominant hand more important than non-dominant.		
Boecker 1999	8 PD, 8 HD, 8 HC	DD 5.87 (3-9) years. H&Y 2.19 (1.5-3). UIII 14.125 (10-20).	S1/M1 and lateral premotor cortex, S2, posterior cingulate (all c/l), prefrontal cortex (b/l) decreased c/w controls. S1, S2 and insula (all i/l) increased c/w controls.	Results suggesting adaptive reorganization to less affected hemisphere.	High-frequency vibratory stimulation applied to the immobilized metacarpal joint of the index finger	PET
Seiss 2003	8 PD, 8 HD, 8 HC	DD 4.25 (1-13) years.	Motor cortex (c/) response normal c/w controls, but S1 (c/) response abnormal (positive deflection in PD, negative in controls at 170ms)		Passive index finger movements + median nerve electrical stim.	EEG (SEP)

Basal Ganglia networks involved in Proprioception from Parkinson's patients

Author Year	No. Subjects	Disease Severity	Brain area involved	Other finding/comment	Task	Interrogation modality
Stefani 2002	9 PD	DD 7-15 years. H&Y ≥ 3.	STN (c/l) important. *GPe (c/l) not important.*	Apomorphine modulates STN response to proprioception (reduced firing with passive movement).	Passive fast (<1s) flexion or extension of the contralateral elbow/wrist	Microelectrode recordings
Kalmar 2011	10 PD, 5 HC	DD 6.5 (2-13) years. H&Y 1.65 (1-3).	Putamen (i/l) increased only in R handed PD patients.	Results suggesting adaptive reorganization to less affected hemisphere.	Passive flexion-extension of the fingers	fMRI
Galazky 2019	3 PD, 6 PSP	PD UIII 46 mean. PSP UIII 24 mean.	PPN (unclear side)	PPN highest firing rate with passive movement c/w active movement=rest	Passive and active movement of ankle flexion/extension (b/l tested at once)	Microelectrode recordings
Boecker 1999	8 PD, 8 HD, 8 HC	DD 5.87 (3-9) years. H&Y 2.19 (1.5-3). UIII 14.125 (10-20).	Globus Pallidus (Putamen trend only) (c/l) had decreased activation c/w controls		High-frequency vibratory stimulation applied to the immobilized metacarpal joint of the index finger	PET

Multisensory Integration at the basal ganglia in non-Parkinsonian animal model

Author/ Year	Basal Ganglia finding	Comment	Sensory test	Animal	Interrogation modality
Nagy 2006	Caudate: 42% of neurons responded to one modality only (17% visual, 6% auditory, 19% tactile). Of the remaining 58%, 19% responded only to 2-3 modalities when isolated, but 39% responded to mixed stimuli. Of the mixed stimuli response, 72% was enhancement of the additive of the two modes of stimuli, and the remainder a depression of the response. The Substantian Nigra was very similar, although with a higher proportion responding to one modality only (75%).	Neurons with the weakest unimodel response had the strongest multimodal effect. Latencies to a multisensory response were actually shorter than unimodal.	Light spots for 1 second with optimum moving direction and velocity for each neuronal unit. White noise at 60db for 1 second. Light tactile stimulation 1cm2 on shaved trunk for 1 second.	Cats.	Microelectrode recording

Multisensory Integration at the basal ganglia in Parkinsonian animal model

Author/ Year	Basal Ganglia finding	Comment	Sensory test	Animal model	Interrogation modality
Rothblat 1995	More response of Gpi and Gpe neurons to mutiple stimulation types in the Parkinsonian model, with reduced response to auditory and visual stimulation.		Multifrequency click, red light, probing face of animal with wooden probe then using von Frey hairs on an electromechanical stimulator to get more precise responses, brushing tail stimulation, punctate stimulation of tail	MPTP model of Parkinsonism on cats.	Microelectrode recording

3. Data extracted from the search strategy for the brain areas active during the mixed proprioceptive and tactile SEP response in healthy and Parkinsonian models.

Cortical networks involved in mixed proprioceptive and tactile response from SEP in PD

Author Year	Sample	PD Severity	PD SEP abnormality	Changes with medication	Changes with DBS	Other/Comment	Task	Interrogation modality
Seiss 2003	8 PD, 8 HC, 8 HD	DD 4.25 (1-13) years.	*N30 equiv between PD and HC*			*Testing was done with patient's normal medication regime on board, and they had low rigidity scores. This might be why N30 scores were normal between PD and HC.*	Median nerve transcutaneous electrical stim.	EEG (SEP)
Rossini 1989	16 PD, 12 HC, 6 other neurol. illness	DD 2.81 (0.4-12) years. H&Y 1-4	Reduced N30 amplitude			• The depressed N30 amplitude in PD was also found in a patient with a tumour of the falx compressing the left SMA.<br>• SMA may be strongly connected to the basal ganglia.	Median nerve transcutaneous electrical stim.	EEG (SEP)
Rossini 1991	35 PD, 30 HC	DD 8 years mean. H&Y 2.22 (1-4)	Reduced N30 amplitude + exaggerated long latency response			?Reduced N30 at complete relaxation in PD due to increased resting tone & therefore ongoing proprioceptive stimulation/gating.	Median nerve transcutaneous electrical stim.	EEG (SEP)

Nakashima 1992	18 PD, 13 HC	H&Y 2.8 (2-3)	*N30 equiv between PD and HC,* however lack of suppression and/or bigger facilitation in recovery curve of central SEP correlated wit increased sensory complaints in PD			A reduced inhibitory function may contribute to sensory disturbance in PD patients. This basal ganglia inhibition may contribute to central SEP amplitude recovery curves.	Median nerve transcutaneous electrical stim.	EEG (SEP)
Mauguiere 1993	7 PD, 25 HC	DD 12.9 ± 3 years	*N30 equiv between PD and HC (except 1/7 PD pt)*	*No change with apomorphine despite improvement in BK and Rigidity*		PD cohort was chronically treated with apomorphine, which could explain the lack of difference in the N30 both "off" and "on" apo acutely (i.e. lingering normalization).	Median nerve transcutaneous stim. before and after apomorphine	EEG (SEP)
Rossini 1993	32 PD, 35 HC	DD 4.8 ± 4.7 years	Reduced N30 amplitude	Apomorphine increased N30 in iPD, most of whom also improved clinically, but did not change parietal component (P14-N20-P25)			Median nerve transcutaneous stim. before and after apomorphine	EEG (SEP)
Babiloni 1994	16 PD, 12 HC	Not stated	Reduced N30 amplitude			Statistical analyses suggest N30 independent from parietal response	Median nerve transcutaneous electrical stim.	EEG (SEP)
Cheron 1994	17 PD (motor fluct.), 10 PD (early), 10 PD mimics, 13 HC	Not stated	Reduced N30 amplitude	Apomorphine increased N30 in iPD (but not for PD mimics with reduced N30), associated with clinical improvement.			Median nerve transcutaneous stim. before and after apomorphine	EEG (SEP)

				N30 improvement correlated with PD severity.<br>No change with apomorphine to other cortical or subcortical SEP components.				
deMari 1995	20 PD	No full text avail.		Apomorphine increased N30 amplitude, associated with improved clinical scores, no change to parietal P14-N20 component.			Median nerve transcutaneous stim. before and after apomorphine	EEG (SEP)
Garcia 1995	10 PD, 10 HC	Not stated	*N30 equiv between PD and HC*				Median nerve transcutaneous electrical stim.	EEG (SEP)
Rossini 1995	43 PD, 17 Parkin-somism, 35 HC	H&Y 2.25 (1-4)	Reduced N30 amplitude + P40 amplitude	Only N30 component improved with apomorphine.			Median nerve transcutaneous stim. before and after apomorphine	EEG (SEP)
Onofrj 1995	40 PD, 40 HC	DD 5 (2-10) years,<br>H&Y 1-4	Reduced N30 amplitude	Not affected by acute or chronic Ldopa or bromocriptime.		Not correlated with dystonia, dyskinetic state, severity on UPDRSIII or predict response to treatment.	Median nerve transcutaneous stim. Acute and chronic response to Ldopa and bromocriptine assessed.	EEG (SEP)
Traversa 1996	33 PD, 11 HC, 6 psychotic with EPSEs	No full text avail.	Reduced N30 amplitude	APO and chronic Ldopa			Median nerve transcutaneous stim. Chronic response to Ldopa and acute response to apomorphine assessed.	EEG (SEP)

Drory 1998	14 PD, 10 HC	DD 6 (2-12) years, UIII 28 (10-50)	Reduced N30 amplitude. N20 response normal.				Median nerve transcutaneous electrical stim.	EEG (SEP)
Insola 1999	2 PD	DD 4 & 10 years	Reduced N30 amplitude		VIM DBS stimulation partially restored N30 amplitude, correlated with control of extrapyramidal symptoms (slight improvement in rigidity).	2 subjects only (both Parkinson's)	Median nerve transcutaneous electrical stim. before and after VIM DBS	EEG (SEP)
Pierantozzi 1999	6 PD	DD 11.8 (5-16) years. UIII 73.9 ± 10.2, H&Y 4.16 (3-5)		Apomorphine increased N30 activity, associated with improved clinical scores	• Gpi or STN DBS: N30 amplitude sig augmentation, which fades when turned off.<br>• Nil changes to parietal (N30, P25) components.	6 subjects only (both Parkinson's)	Median nerve transcutaneous electrical stim before and after GPi or STN DBS	EEG (SEP)
Bostantjo-Poulou 2000	23 PD, 23 HC	H&Y 1-2, LEDD 648 +- 75mg.	Reduced N30 amplitude (b/l)<br>Reduced N30-P40 amplitude (R side only)			SPECT suggests N30 associated with parietal, not frontal, blood flow.	Median nerve transcutaneous electrical stim	EEG (SEP) + SPECT CBF

Pierantozzi 2000	9 PD, 9 HC, 7 psychotic EPSE	H&Y 2.8 (2-4), UIII 56.2 ± 16.8		N30 activity increased by peripheral neuromuscular block for PD and Neuroleptic Malignant Syndrome patients, with a small increase for HC.		Suggests that increased rigidity (& perhaps proprioceptive noise) may be the cause of reduced N30 amplitude in PD.	Median nerve transcutaneous electrical stim before and after peripheral neuromuscular block	EEG (SEP)
Bostantjo-Poulou 2002	20 PD	DD 9.8 ± 5.3 years, H&Y 3-4, LEDD 840 ± 264.3		N30 amplitude increased in PD after amantadine (6 day infusion)			Median nerve transcutaneous electrical stim, before and after 6 day course amantadine infusion	EEG (SEP)
Insola 2005	6 PD	Mod-severe (needing DBS)			Parietal component transiently obliterated after DBS surgery (likely shock effect), N30 improvement post STN stimulation	6 subjects only (both Parkinson's)	Median nerve transcutaneous electrical stim. before and after STN DBS	EEG (SEP)
Macerollo 2016	18 PD, 18 HC	DD 4.22 ± 2.36 years, UIII 11.83 ± 3.69	Reduced N20-P25 amplitude	Improved to normal with antiparkinsonian medication.			Median nerve transcutaneous electrical stim, on and off medication	EEG (SEP)
Tinazzi 1999	14 PD, 16 HC	DD 4.07 (1.8-7) years	Reduced P37-N50 (vertex-central) amplitude, correlated with clinical severity. *Brainstem P30 and frontal N37*	• Apomorphine increased P37-N50 amplitude, correlated with clinical improvement.<br>• Apomorphine did not alter other SEPs.		Hypothesized that low amplitude of P37-N50 may be due to improper modulation of cortical excitability by the basal ganglia, causing abnormal processing of	Tibial nerve transcutaneous electrical stim., on and off medication	EEG (SEP)

			equivalent to controls.			somatosensory inputs in the frontal lobe.		
Mochizuki 1999	17 PD, 20 HC, 3 Epilepsy	Not stated	Enlarged High Frequency Oscillations (HFOs) at N20 (area 3b of S1) in PD			In PD dysfunction of the basal ganglia may influence the excitability of thalamocortical projection neurons, leading to abnormally enlarged repetitive discharges in S1, leading to enlarged HFOs	Median nerve transcutaneous electrical stim., on and off medication	EEG (SEP)
Inoue 2001	5 PD, 9 HC, 6 MSA	DD 5.5 ± 5.3 years	Enlarged High Frequency Oscillations (HFOs) at P37 (vertex-central) in PD				Tibial nerve transcutaneous electrical stim., on and off medication	EEG (SEP)
Machii 2003	5 PD, 7 HC, 5 ALS/PD, 5 ALS	Not stated	● *SEPs all equivalent b/w groups.*<br>● *Second pulse at long ISI not suppressed in PD or HC, c/w ALS/PD.*			*Normal second pulse amplitude suggests PD intracortical inhibition is normal, despite the findings of other studies.*	Median nerve transcutaneous electrical stim., on and off medication	EEG (SEP)
Weise 2015	50 PD, 50 HC	Not stated	*Auricular Branch of Vagus Nerve (ABVN) SEP equivalent amplitude and latency between PD and HC Trigeminal SEP equivalent*				Transcutaneous electric stimulation of ABVN (tragus) & Transcutaneous electrical stim. of trigeminal nerve of upper and lower lip.	EEG (SEP)

			latency (amplitude not measured) between PD and HC					
Seiss 2003	8 PD, 8 HC, 8 HD	DD 4.25 (1-13) years	*N30 equiv between PD and HC*			Testing was done with patient's normal medication regime on board, and they had low rigidity scores. This might be why N30 scores were normal between PD and HC.	Median nerve transcutaneous electrical stim.	EEG (SEP)

Basal ganglia networks involved in mixed proprioceptive and tactile response from SEP in PD

Author Year	Sample	PD Severity	PD SEP abnormality	Other/Comment	Task	Interrogation modality
Pesenti 2003	8 PD	UIII 41.7 ± 10.1,<br>LEDD 1290 ± 495	• P/N18 component detected at STN 1.6 msec before N20 parietal SEP<br>• Tactile cutaneous stimulation failed to evoke SEPs from both the scalp and the STN	• Sensory (mixed proprioceptive/tactile) network involves STN.<br>• The pair of contacts used to record STN SEPs are very close (2 mm), and this makes very unlikely the possibility of far field contamination of the signal.<br>• Failure of pure tactile response may be due to cutaneous afferents not bypassing STN, unlike muscle afferents	Median nerve transcutaneous electrical stim. at the wrist (tactile and proprioceptive), and via ring electrodes (pure tactile).	Microelectrode recording

				from a median mixed nerve response (proprioception).		
Tsai 2015	8 PD	Full text not avail.	During Median Nerve Stimulation STN had reduced bursting pattern, more beta band oscillation, more power spectral density	Sensory (mixed proprioceptive/tactile) network involves STN	Median nerve transcutaneous electrical stim.	Microelectrode recording
Trenado 2017	12 PD	DD 12.2 ± 4.6 years	At STN able to record four distinctive long latency SEPs (P80, N100, P140, N200)	Sensory (mixed proprioceptive/tactile) network involves STN	Median nerve transcutaneous electrical stim.	Microelectrode recording

Cortical networks involved in mixed proprioceptive and tactile response from SEP in PD animal models

Author Year	PD SEP abnormality	Changes with medication	Other/Comment	Animal model	Task	Interrogation modality
Onofrj 1994	Reduced N15 amplitude	• Not reversed with Levodopa despite clinical improvement.<br>• Increased amplitude with anaesthetics NMDA and etomidate (GABAergic anaesthetic)	• N15 in monkeys may be similar to N30 in humans.<br>• Decreased in N15 independent of severity of symptoms.<br>• ?improvement to GABAergic anaesthetic similar to improvement post peripheral neuromuscular blockade.	MPTP model in cynomolgus monkeys.	Median nerve transcutaneous electrical stim.	EEG (SEP)

4. Somatopic organisation of nuclei in the basal ganglia

Somatopic organization of nuclei in the basal ganglia demonstrated with microelectrode recordings with passive movement (proprioception) in Parkinson's patients

Author/Year	Somatopy (gradient)	Responding to single/multiple	Other findings/Comment
Sterio 1994	GP: Somatopically arranged cell clusters identified.	Neurons usually responded soley to a single joint movement.	• Different patterns of activity for moving that joint in different directions.<br>• Less somatopic than in previous primate studies ?Because of reduced specificity in human Parkinsons patients
Taha 1996	GPi: Arm cells rostral and caudal ends with Leg cells central.	Neurons responded more to single (68%) rather > multiple (32%) joints	• Neurons responded to more proximal (75%) > distal (25%) joints, however with more distal joint response in GPi>Gpe.<br>• More kinesthetic cells were activated (63%) > inhibited (28%).
Theodosopoulos 2003	• Dorsal STN for all responsive neurons.<br>• STN: Arm: Laterally, at rostral and caudal poles.<br>Leg: medial and central.	75% responded to single joint, <5% responded to movement of multiple limbs.	• Neurons responded to more proximal (shoulder, elbow, hip or knee) > distal (wrist or ankle) joints.<br>• Arm responses > leg responses. Arm responses more widespread.
Romanelli 2004	STN: Arm: Lateral, dorsal and posterior.<br>Leg: Medial, ventral and anterior.	12% responded to both arm and leg movements.	Leg cells (46%) > arm cells (40%) > orofacial cells (2%)

Zaidel 2010	Dorsolateral (62%) > ventral (25%) STN response.		
Sasaki 2019	● Dorsolateral STN contains most movement responsive cells.<br>● STN: Arm: Lateral, anterior and dorsal.<br>Leg: Medial, posterior and ventral.<br>● Gradient from anterior-dorsal-lateral edge: finger - wrist - elbow - shoulder - ankle- knee - hip.		● The most common STN DBS site was found to be slightly inferior, medial and posterior to the site of maximum proprioceptive responsiveness.<br>● Hypothesized that dorsolateral STN cells connect to M1, and the area of most common STN DBS stimulation projects to SMA.
Boiler 2020	STN: Arm: lateral, anterior, dorsal.<br>Leg: medial, posterior, ventral.		

Somatopic organization of nuclei in the basal ganglia demonstrated with microelectrode recordings with passive and active movement in Parkinson's patients

Author/Year	Somatopy (gradient)	Responding to single/multiple	Other findings/Comment
Rodriguez-Oroz 2001	● Dorsolateral STN contain all movement responsive neurons.<br>● STN: Oromandibular: most ventral.<br>Arm: lateral.<br>Leg: most medial.<br>● Substantia Nigra: 11% responded to sensorimotor stimulation.		● Study pooled active and passive movement data.<br>● Dorsolateral neurons are irregular or tonic type. Ventral (non-responsive) neurons are of oscillatory or low frequency activity. 84% of tremor neurons were sensitive to proprioception.
Baker 2010	GPi: Orofacial neurons were most ventral.<br>Arm: Middle ventral, most lateral, posterior.<br>Leg: Most dorsal, medial, anterior.	85.8% of neurons responded to a single contralateral body region, with 14.2% responding to multiple	● Study pooled active and passive movement data.<br>● Comment that passive movement responses were noted.<br>● Neurons responded to more proximal > distal joints

		and/or ipsilateral body regions.	
Abosch 2002	● Dorsolateral STN 65% of movement responsive cells.<br>● 96.8% in rostral 2/3 of the STN.	76% responsive to single joint, 24% receptive to multiple joints.	Arm cells (32%) > leg cells (21%) > orofacial cells (14%)

Somatopic organization of nuclei in the basal ganglia demonstrated with microelectrode recordings with passive movement proprioception in non-Parkinsonian animals

Author/ Year	Somatopy (gradient)	Responding to single/multiple	Animal	Other findings/Comment
DeLong 1985	● GPe and Gpi: Orofacial: Caudal, most ventral.<br>Arm: All rostro-caudal but more caudally. Inferior (ventral) and lateral to leg.<br>Leg: Central but more rostral than arm and central but more dorsal than arm.<br>● STN: Orofacial: Ventrolateral, throughout rostro-caudal extent.<br>Arm: All rostro-caudal but most at rostro and caudal poles.<br>Leg: Central in rostro-caudal and medial-lateral dimensions.		Monkeys	● Of cells whose discharge was related to active limb movements: 37%, 22% and 20% responded to passive movement in the Gpe, Gpi and STN respectively.<br>● Response usually specific in relation to joint and direction of movement.
Iwamuro 2017	● STN: Orofacial: most lateral and medial.<br>Leg: most central.<br>Arm: in between.<br>● GPe+GPi: Orofacial = ventral.<br>Leg: dorsal.<br>Arm: inbetween	Majority of neurons responding to single body parts, some responding to two adjacent body parts.	Monkeys	● Study maps proprioceptive response via microelectrode recordings of basal ganglia neurons which also respond to SMA or M1 microelectric stimulation.<br>● SMA responsive neurons had less proprioceptive response (none in GPe/GPi).<br>● Most STN and GPe/GPi neurons responded exclusively to stimulation of either the MI or

				SMA, and 1/4 to 1/3 of neurons responded to both.

COMMENT: Rodriguez-Oroz (2001) found that dorsolateral neurons were of the irregular or tonic type, and non-responding ventral neurons were of oscillatory or low-frequency activity. Sasaki (2019) found the most common site of STN DBS stimulation in Parkinson's to be slightly inferior, medial and posterior to the site of maximum proprioceptive responsiveness. They hypothesized that this was because the most efficient site for DBS stimulation is over cells that project to the SMA, and the proprioception responsive cells likely project to M1. Indeed, Iwamuro (2017) found SMA responsive neurons less likely to respond to proprioception than M1 responsive neurons. There was however overlap, with 25% of STN neurons and 33% of GPe/GPi neurons responding to both SMA and M1. Sasaki's hypothesis, if confirmed, would be evidence against the possibility that direct modulation of proprioceptive abnormality in Parkinson's is what results in the greatest motoric benefit. Rodriguez-Oroz (2001) however, did find that 84% of tremor neurons were sensitive to proprioception.

5. Studies demonstrating sensorimotor integration in Parkinson's disease

Author Year	Sensorimotor Integration Finding	Other finding/comment	Task	Interrogation modality
Tamburin 2003	Decreased tactile sensory gating of motor	Reduced intracortical inhibition	Electrical stimulation via ring electrodes to digit 2 or 5	TMS
Schrader 2008	Decreased proprioceptive sensory gating of motor	Reduced intracortical inhibition	Muscle vibration at Extensor Carpi Radialis	TMS
Vinding 2019	Decreased proprioceptive sensory gating of motor	Reduced intracortical inhibition	Precisely controlled passive movement of index finger, measuring FCR to make sure no active muscle activity	MEG (ERP)
Degardin 2009	Decreased proprioceptive sensory gating of motor	Reduced intracortical inhibition. Levodopa reversed this effect after active, but not passive movement or transcutaneous electrical median nerve stimulation.	Passive and active index finger movement and transcutaneous electrical median nerve stimulation.	EEG (ERP)

Rossini 1991	Decreased tactile & proprioceptive sensory gating of motor	Reduced intracortical inhibition. Largest effect in iPD with depressed frontal N30.	Median or ulnar nerve transcutaneous stimulation.	TCS
Cheron 1994	*Preserved motor gating of sensory function*	Despite lower N30 component at rest, both when ON and OFF apomorphine	Median nerve transcutaneous stim. & Voluntary movements (clenching of hand).	EEG (SEP)
Macerollo 2016	Decreased motor gating of sensory function	Reduced intracortical inhibition. Attenuation returned when on medication. Amount of attenuation inversely correlated with UPDRSIII scores.	Median nerve transcutaneous stim. & Voluntary movements (abduction of R thumb).	EEG (SEP)

COMMENT: Rocchi (2017) showed increased inhibition in S1 with paired pulse somatosensory evoked potentials (SEPs) and high frequency oscillations (HFOs) after transcranial magnetic stimulation (TMS) motor (M1) stimulation. Rossini (1991) showed, in healthy controls, a reduced frontal (N30), parietal (P20) SEP response and increased long latency SEP response (LLRs) at the time of maximal muscle contraction. Marcerollo (2016) demonstrated reduced amplitude of the parietal SEP component with concurrent voluntary movement at the same site as sensory stimulation, suggesting increased S1 inhibition with motor activity. Lei (2018) revealed decreased amplitude of the N20/P25 response, thought to originate from S1, at the time of increased motor activity at the same site as sensory stimulation. Lei also demonstrated that a cortically-detected subcortical component of the SEP, felt to arise from between the medial lemniscus and thalamus, had decreased amplitude during the same motor activity, suggesting that this gating form of sensorimotor integration also occurs at a level inferior to the thalamus, potentially even at the Basal Ganglia. Tamburin (2003) showed this via a reduced amplitude TMS over M1 induced motor evoked potential (MEP) at the time of tactile stimulation via ring electrodes in the same body part as the MEP. Schrader (2008) demonstrated this via reduced MEP at the time of proprioceptive (muscle vibratory) stimulation. The Tamburin (2003) and Schrader (2008) studies mentioned above also revealed, in Parkinson's patients compared to healthy controls, reduced inhibition of motor responses after proprioceptive or tactile stimulation. Macerollo (2016) found the normal inhibition of the S1 SEP response at onset of voluntary movement was reduced in Parkinson's patients when off dopaminergic medication, and the degree of reduction in inhibition correlated with Parkinson's severity. The reduced inhibition was found to partially normalise when dopaminergic medication was reinstated. Rossini (1991) found long latency SEP responses (LLRs) at rest in Parkinson's patients, that were not seen in healthy controls. LLRs were correlated with degree of N30 amplitude reduction, and voluntary muscle activation increased LLRs further. Voluntary muscle activation also caused a small amplitude reduction in the S1 or parietal N20 component, but a complete flattening of the N30. LLRs therefore may be hypothesized to be brain neuronal activity related to high resting muscle tone in Parkinson's patients, and it may be this pathophysiology that is causing reduction

in amplitude of the N30 SEP component via a gating mechanism. Indeed, Rossini found a weak association between these findings and bradykinesia and rigidity scores in Parkinson's patients. Degardin (2009) and Vinding (2019) showed with magnetoencephalogram (MEG) (Vindig, 2019) and EEG (Degardin, 2009) a reduction in the normal beta rebound (synchronization), originating from S1 and M1, which follows desynchronization in S1 to proprioceptive stimulus. Because motor commands are inhibited during the synchronization phase (sensorimotor gating), Parkinsonian patients therefore have reduced inhibition of movement in the second phase after proprioceptive stimulation. Degardin (2009) found dopaminergic medication unable to normalize the impaired sensorimotor gating in Parkinson's.

6. Studies demonstrating increased noise / decreased specificity at the basal ganglia in Parkinson's disease

Human studies showing increase noise / decreased specificity in the Basal Ganglia in Parkinson's at resting state, without sensory testing.

Author/Year	Finding	Comment	Interrogation modality
Steigerwald 2008	Significant increase in the mean firing rate of STN neurons in PD patients and a relatively larger fraction of neurons exhibiting burst-like activity compared with ET patients	As per Remple (2011) mean firing rate increases the background STN noise (root mean square of all STN recording).	Microelectrode recording
Remple 2011	Background STN noise (root mean square of all STN recordings) was significantly lower in early vs late PD.		Microelectrode recording
Vyas 2016	STN: No sig differences in firing rate but reduced neuronal "complexity"	Consistent with studies correlating loss of variability in neuronal activity to disease state	Microelectrode recording

Animal studies showing increase noise / decreased specificity in the Basal Ganglia in Parkinson's at resting state, without sensory testing.

Author/ Year	Basal Ganglia finding	Cortical finding	Comment	Animal model	Interrogation modality
Pelled 2005	Reduction in temporal and spatial variance in left and right striatum of PD group.	Reduction in temporal and spatial variance in left and right sensorimotor cortices of PD group.	No temporal variance change in control areas (substantia innominata, muscular region outside the brain).	6-OHDA lesioning of dopamine neurons on rats.	RS-fMRI
Mallet 2008	GP: 100 fold increase in synchronization of GP units during beta oscillations, however mean firing rate decreased. Neurons of same type fired together, with small phase differences. Changes persisted across extreme brain states.		Increased synchronization, like-neurons firing together with small phase differences = decreased differentiation/increased noise.	6-OHDA lesioning of dopamine neurons on rats.	Microelectrode recording

Animal studies utilising sensory testing showing increased noise / decreased specificity in Parkinson's disease models

Author/ Year	Basal Ganglia finding	Cortical finding	Comment	Animal model	Interrogation modality
Escola 2002		With increasing PD: increasing pre-SMA responsiveness to more than one joint (at the extreme responding to UL and LL joints). SMAp response also becomes less specific.	With increasing PD severity, increasing pre-SMA responsiveness to passive movement.	MPTP model of Parkinsonism on primates.	Microelectrode recording
Rothblat 1995	Parkinsonian cats had larger receptive fields to tactile stimulation in GPi and GPe and more bilateral receptive fields.			MPTP model of Parkinsonism on cats.	Microelectrode recording
Erez 2011	With parkinsonian state, more neurons responded to the same passive movement (proprioception), although they responded differently.		Suggestion this supports the "loss of specificity" finding in other studies.	MPTP model of Parkinsonism on primates.	Microelectrode recording

Pessiglione 2005	With parkinsonian state, receptive fields for proprioception in pallidonigral thallamus increased, most of this due to increased neurons that responded to 2 or more limbs.			MPTP model of Parkinsonism on primates.	Microelectrode recording

COMMENT: Pessiglione (2005) demonstrated that there was a significant increase, in the Parkinsonian state, of neurons responding to proprioceptive stimulation in two or more limbs. Erez (2011) demonstrated a more widespread neuronal response to the same proprioceptive stimulus. This may be due to an increase in receptive field size at the STN, which has been demonstrated for proprioception (Pessiglione, 2005) and for tactile stimulation (Rothblat, 1995). Indeed, the Rothblat (1995) finding showed this decreased specificity and increased receptive field size even applies across different sensory modalities, with GPi and GPe neurons responding more frequently to multiple different sensory modalities (auditory, visual, tactile) in the Parkinsonian model.

Pelled (2005) demonstrated this as reduction in temporal and spatial variance in the striatum in the Parkinsonian rat, after 6-OHDA lesioning. Mallet (2008) revealed a 100 fold increase in synchronization of globus pallidus neuronal units, equating to a loss of specificity. A human study (Vyas, 2016) was supportive of this, finding a reduction in neuronal activity complexity in Parkinsonism. Steigerwald (2008) and Remple (2011) demonstrated via microelectrode recordings in humans at the STN, the resting state mean neuronal firing rate was significantly increased. This was referred to as "noise" by Remple, with a larger fraction of neurons exhibiting burst-like activity (Steigerwald, 2008).

Increasing Parkinsonism resulted increasing pre-SMA and SMAp neuron responsiveness to proprioception at more than one joint. At the extreme a single neuron responded to passive movements of both upper and lower limb joints.

References

1. Abosch, A., Hutchison, W. D., Saint-Cyr, J. A., Dostrovsky, J. O. & Lozano, A. M. Movement-related neurons of the subthalamic nucleus in patients with Parkinson disease. *Journal of neurosurgery* **97**, 1167–72 (2002).
2. Akatsuka, K. *et al.* Mismatch responses related to temporal discrimination of somatosensory stimulation. *Clinical neurophysiology : official journal of the International Federation of Clinical Neurophysiology* **116**, 1930–7 (2005).
3. Babiloni, F. *et al.* Statistical analysis of topographic maps of short-latency somatosensory evoked potentials in normal and parkinsonian subjects. *IEEE transactions on bio-medical engineering* **41**, 617–24 (1994).
4. Baker, K. B. *et al.* Somatotopic organization in the internal segment of the globus pallidus in Parkinson's disease. *Experimental neurology* **222**, 219–25 (2010).
5. Baumgarten, T. J., Konigs, S., Schnitzler, A. & Lange, J. Subliminal stimuli modulate somatosensory perception rhythmically and provide evidence for discrete perception. *Scientific reports* **7**, 43937 (2017).
6. Baumgarten, T. J., Schnitzler, A. & Lange, J. Beta oscillations define discrete perceptual cycles in the somatosensory domain. *Proceedings of the National Academy of Sciences of the United States of America* **112**, 12187–92 (2015).
7. Boecker, H. *et al.* Sensory processing in Parkinson's and Huntington's disease: investigations with 3D H(2)(15)O-PET. *Brain : a journal of neurology* **122 (Pt 9)**, 1651–65 (1999).
8. Bolier, E. *et al.* Kinesthetic Cells within the Subthalamic Nucleus and Deep Brain Stimulation for Parkinson Disease. *World neurosurgery* **139**, e784–e791 (2020).
9. Bolognini, N., Papagno, C., Moroni, D. & Maravita, A. Tactile temporal processing in the auditory cortex. *Journal of cognitive neuroscience* **22**, 1201–11 (2010).
10. Bostantjopoulou, S., Katsarou, Z., Georgiadis, G., Zafiriou, D. & Kazis, A. Amantadine sulfate infusion effect on N30 somatosensory evoked potentials in Parkinson's disease. *Clinical neuropharmacology* **25**, 115–8 (2002).

11. Bostantjopoulou, S. *et al.* Abnormality of N30 somatosensory evoked potentials in Parkinson's disease: a multidisciplinary approach. *Neurophysiologie clinique = Clinical neurophysiology* **30**, 368–76 (2000).

12. Bueti, D., Bahrami, B. & Walsh, V. Sensory and association cortex in time perception. *Journal of cognitive neuroscience* **20**, 1054–62 (2008).

13. Cardoso, E. F. *et al.* Abnormal visual activation in Parkinson's disease patients. *Movement Disorders* **25**, 1590–6 (2010).

14. Cheron, G., Piette, T., Thiriaux, A., Jacquy, J. & Godaux, E. Somatosensory evoked potentials at rest and during movement in Parkinson's disease: evidence for a specific apomorphine effect on the frontal N30 wave. *Electroencephalography and clinical neurophysiology* **92**, 491–501 (1994).

15. Conte, A. *et al.* Understanding the link between somatosensory temporal discrimination and movement execution in healthy subjects. *Physiological reports* **4**, (2016).

16. Conte, A. *et al.* Subthalamic nucleus stimulation and somatosensory temporal discrimination in Parkinson's disease. *Brain : a journal of neurology* **133**, 2656–63 (2010).

17. Conte, A. *et al.* Theta-burst stimulation-induced plasticity over primary somatosensory cortex changes somatosensory temporal discrimination in healthy humans. *PloS one* **7**, e32979 (2012).

18. de Mari, M., Margari, L., Lamberti, P., Iliceto, G. & Ferrari, E. Changes in the amplitude of the N30 frontal component of SEPs during apomorphine test in parkinsonian patients. *Journal of neural transmission. Supplementum* **45**, 171–6 (1995).

19. Degardin, A. *et al.* Deficient 'sensory' beta synchronization in Parkinson's disease. *Clinical neurophysiology : official journal of the International Federation of Clinical Neurophysiology* **120**, 636–42 (2009).

20. DeLong, M. R., Crutcher, M. D. & Georgopoulos, A. P. Primate globus pallidus and subthalamic nucleus: functional organization. *Journal of neurophysiology* **53**, 530–43 (1985).

21. Di Biasio, F. *et al.* Does the cerebellum intervene in the abnormal somatosensory temporal discrimination in Parkinson's disease? *Parkinsonism & related disorders* **21**, 789–92 (2015).

22. Drory, V. E., Inzelberg, R., Groozman, G. B. & Korczyn, A. D. N30 somatosensory evoked

potentials in patients with unilateral Parkinson's disease. *Acta neurologica Scandinavica* **97**, 73–6 (1998).

23. Dusek, P. *et al.* Abnormal activity in the precuneus during time perception in Parkinson's disease: an fMRI study. *PloS one* **7**, e29635 (2012).
24. Elsinger, C. L. *et al.* Neural basis for impaired time reproduction in Parkinson's disease: an fMRI study. *Journal of the International Neuropsychological Society : JINS* **9**, 1088–98 (2003).
25. Erez, Y., Tischler, H., Belelovsky, K. & Bar-Gad, I. Dispersed activity during passive movement in the globus pallidus of the 1-methyl-4-phenyl-1,2,3,6-tetrahydropyridine (MPTP)-treated primate. *PloS one* **6**, e16293 (2011).
26. Escola, L. *et al.* Disruption of the proprioceptive mapping in the medial wall of parkinsonian monkeys. *Annals of neurology* **52**, 581–7 (2002).
27. Ferrandez, A. M. *et al.* Basal ganglia and supplementary motor area subtend duration perception: an fMRI study. *NeuroImage* **19**, 1532–44 (2003).
28. Galazky, I. *et al.* Neuronal spiking in the pedunculopontine nucleus in progressive supranuclear palsy and in idiopathic Parkinson's disease. *Journal of neurology* **266**, 2244–2251 (2019).
29. Garcia, P. A., Aminoff, M. J. & Goodin, D. S. The frontal N30 component of the median-derived SEP in patients with predominantly unilateral Parkinson's disease. *Neurology* **45**, 989–92 (1995).
30. Ghilardi, M. F., Bodis-Wollner, I., Onofrj, M. C., Marx, M. S. & Glover, A. A. Spatial frequency-dependent abnormalities of the pattern electroretinogram and visual evoked potentials in a parkinsonian monkey model. *Brain* **111 (Pt 1)**, 131–49 (1988).
31. Hannula, H. *et al.* Navigated transcranial magnetic stimulation of the primary somatosensory cortex impairs perceptual processing of tactile temporal discrimination. *Neuroscience letters* **437**, 144–7 (2008).
32. Huang, C. W., Lin, C. H., Lin, Y. H., Tsai, H. Y. & Tseng, M. T. Neural Basis of Somatosensory Spatial and Temporal Discrimination in Humans: The Role of Sensory Detection. *Cerebral cortex (New York, N.Y. : 1991)* **32**, 1480–1493 (2022).
33. Inoue, K., Hashimoto, I. & Nakamura, S. High-frequency oscillations in human posterior tibial

somatosensory evoked potentials are enhanced in patients with Parkinson's disease and multiple system atrophy. *Neuroscience letters* **297**, 89–92 (2001).

34. Insola, A., Mazzone, P. & Valeriani, M. Somatosensory evoked potential and clinical changes after electrode implant in basal ganglia of parkinsonian patients. *Muscle & nerve* **32**, 791–7 (2005).
35. Insola, A., Rossi, S., Mazzone, P. & Pasqualetti, P. Parallel processing of sensory inputs: an evoked potentials study in Parkinsonian patients implanted with thalamic stimulators. *Clinical neurophysiology : official journal of the International Federation of Clinical Neurophysiology* **110**, 146–51 (1999).
36. Iwamuro, H., Tachibana, Y., Ugawa, Y., Saito, N. & Nambu, A. Information processing from the motor cortices to the subthalamic nucleus and globus pallidus and their somatotopic organizations revealed electrophysiologically in monkeys. *The European journal of neuroscience* **46**, 2684–2701 (2017).
37. Kalmar, Z. *et al.* Reorganization of motor system in Parkinson's disease. *European neurology* **66**, 220–6 (2011).
38. Karim, A. A., Schüler, A., Hegner, Y. L., Friedel, E. & Godde, B. Facilitating effect of 15-Hz repetitive transcranial magnetic stimulation on tactile perceptual learning. *J Cogn Neurosci* **18**, 1577–1585 (2006).
39. Koch, G. *et al.* High-frequency rTMS improves time perception in Parkinson disease. *Neurology* **63**, 2405–6 (2004).
40. Kupersmith, M. J., Shakin, E., Siegel, I. M. & Lieberman, A. Visual system abnormalities in patients with Parkinson's disease. *Arch Neurol* **39**, 284–6 (1982).
41. Lacruz, F., Artieda, J., Pastor, M. A. & Obeso, J. A. The anatomical basis of somaesthetic temporal discrimination in humans. *Journal of neurology, neurosurgery, and psychiatry* **54**, 1077–81 (1991).
42. Lei, Y., Ozdemir, R. A. & Perez, M. A. Gating of Sensory Input at Subcortical and Cortical Levels during Grasping in Humans. *The Journal of neuroscience : the official journal of the Society for Neuroscience* **38**, 7237–7247 (2018).

43. Leodori, G. *et al.* The third-stimulus temporal discrimination threshold: focusing on the temporal processing of sensory input within primary somatosensory cortex. *Journal of neurophysiology* **118**, 2311–2317 (2017).

44. Macerollo, A. *et al.* Dopaminergic treatment modulates sensory attenuation at the onset of the movement in Parkinson's disease: A test of a new framework for bradykinesia. *Movement disorders : official journal of the Movement Disorder Society* **31**, 143–6 (2016).

45. Machii, K., Ugawa, Y., Kokubo, Y., Sasaki, R. & Kuzuhara, S. Somatosensory evoked potential recovery in kii amyotrophic lateral sclerosis/parkinsonism-dementia complex (kii AlS/PDC). *Clinical neurophysiology : official journal of the International Federation of Clinical Neurophysiology* **114**, 564–8 (2003).

46. Mallet, N. *et al.* Parkinsonian beta oscillations in the external globus pallidus and their relationship with subthalamic nucleus activity. *The Journal of neuroscience : the official journal of the Society for Neuroscience* **28**, 14245–58 (2008).

47. Manzo, N. *et al.* Investigating the effects of transcranial alternating current stimulation on primary somatosensory cortex. *Scientific reports* **10**, 17129 (2020).

48. Mauguiere, F., Broussolle, E. & Isnard, J. Apomorphine-induced relief of the akinetic-rigid syndrome and early median nerve somatosensory evoked potentials (SEPs) in Parkinson's disease. *Electroencephalography and clinical neurophysiology* **88**, 243–54 (1993).

49. Mochizuki, H. *et al.* Somatosensory evoked high-frequency oscillation in Parkinson's disease and myoclonus epilepsy. *Clinical neurophysiology : official journal of the International Federation of Clinical Neurophysiology* **110**, 185–91 (1999).

50. Mowery, T. M., Harrold, J. B. & Alloway, K. D. Repeated whisker stimulation evokes invariant neuronal responses in the dorsolateral striatum of anesthetized rats: a potential correlate of sensorimotor habits. *Journal of neurophysiology* **105**, 2225–38 (2011).

51. Nagy, A., Eordegh, G., Paroczy, Z., Markus, Z. & Benedek, G. Multisensory integration in the basal ganglia. *The European journal of neuroscience* **24**, 917–24 (2006).

52. Nakashima, K., Nitta, T. & Takahashi, K. Recovery functions of somatosensory evoked potentials in parkinsonian patients. *Journal of the neurological sciences* **108**, 24–31 (1992).

53. Nenadic, I. *et al.* Processing of temporal information and the basal ganglia: new evidence from fMRI. *Experimental brain research* **148**, 238–46 (2003).

54. Okuda, B., Tachibana, H., Takeda, M., Kawabata, K. & Sugita, M. Visual and somatosensory evoked potentials in Parkinson's and Binswanger's disease. *Dementia (Basel, Switzerland)* **7**, 53–8 (1996).

55. Onofrj, M., Ferracci, F., Fulgente, T., Malatesta, G. & Ghilardi, M. F. Effects of drug manipulations on anterior components of somatosensory evoked potentials in a parkinsonian animal model. *Drugs under experimental and clinical research* **20**, 29–36 (1994).

56. Onofrj, M. *et al.* The abnormality of N30 somatosensory evoked potential in idiopathic Parkinson's disease is unrelated to disease stage or clinical scores and insensitive to dopamine manipulations. *Movement disorders : official journal of the Movement Disorder Society* **10**, 71–80 (1995).

57. Otsuru, N. *et al.* 10 Hz transcranial alternating current stimulation over posterior parietal cortex facilitates tactile temporal order judgment. *Behavioural brain research* **368**, 111899 (2019).

58. Palomar, F. J. *et al.* Sensory perception changes induced by transcranial magnetic stimulation over the primary somatosensory cortex in Parkinson's disease. *Movement Disorders* **26**, 2058–64 (2011).

59. Pastor, M. A., Day, B. L., Macaluso, E., Friston, K. J. & Frackowiak, R. S. The functional neuroanatomy of temporal discrimination. *The Journal of neuroscience : the official journal of the Society for Neuroscience* **24**, 2585–91 (2004).

60. Pastor, M. A., Macaluso, E., Day, B. L. & Frackowiak, R. S. The neural basis of temporal auditory discrimination. *NeuroImage* **30**, 512–20 (2006).

61. Pekkonen, E., Jousmaki, V., Reinikainen, K. & Partanen, J. Automatic auditory discrimination is impaired in Parkinson's disease. *Electroencephalography and clinical neurophysiology* **95**, 47–52 (1995).

62. Pelled, G., Bergman, H., Ben-Hur, T. & Goelman, G. Reduced basal activity and increased functional homogeneity in sensorimotor and striatum of a Parkinson's disease rat model: a functional MRI study. *The European journal of neuroscience* **21**, 2227–32 (2005).

63. Peppe, A. *et al.* Low contrast stimuli enhance PERG sensitivity to the visual dysfunction in Parkinson's disease. *Electroencephalography and clinical neurophysiology* **82**, 453–7 (1992).
64. Pesenti, A. *et al.* Subthalamic somatosensory evoked potentials in Parkinson's disease. *Movement disorders : official journal of the Movement Disorder Society* **18**, 1341–5 (2003).
65. Pessiglione, M. *et al.* Thalamic neuronal activity in dopamine-depleted primates: evidence for a loss of functional segregation within basal ganglia circuits. *The Journal of neuroscience : the official journal of the Society for Neuroscience* **25**, 1523–31 (2005).
66. Philipova, D., Gatchev, G., Vladova, T. & Georgiev, D. Event-related potentials in parkinsonian patients under auditory discrimination tasks. *International Journal of Psychophysiology* **27**, 69–78 (1997).
67. Pierantozzi, M. *et al.* The effect of deep brain stimulation on the frontal N30 component of somatosensory evoked potentials in advanced Parkinson's disease patients. *Clinical neurophysiology : official journal of the International Federation of Clinical Neurophysiology* **110**, 1700–7 (1999).
68. Pierantozzi, M. *et al.* Curariform peripheral block of muscular tone selectively increases precentral N30 somatosensory evoked potentials component. A pharmacological study carried out on healthy subjects and parkinsonian syndromes. *Experimental brain research* **133**, 368–76 (2000).
69. Rai, N., Premji, A., Tommerdahl, M. & Nelson, A. J. Continuous theta-burst rTMS over primary somatosensory cortex modulates tactile perception on the hand. *Clinical neurophysiology : official journal of the International Federation of Clinical Neurophysiology* **123**, 1226–33 (2012).
70. Remple, M. S. *et al.* Subthalamic nucleus neuronal firing rate increases with Parkinson's disease progression. *Movement disorders : official journal of the Movement Disorder Society* **26**, 1657–62 (2011).
71. Rocchi, L., Casula, E., Tocco, P., Berardelli, A. & Rothwell, J. Somatosensory Temporal Discrimination Threshold Involves Inhibitory Mechanisms in the Primary Somatosensory Area. *The Journal of neuroscience : the official journal of the Society for Neuroscience* **36**, 325–35

(2016).

72. Rocchi, L. *et al.* High frequency somatosensory stimulation increases sensori-motor inhibition and leads to perceptual improvement in healthy subjects. *Clinical neurophysiology : official journal of the International Federation of Clinical Neurophysiology* **128**, 1015–1025 (2017).

73. Rodriguez-Oroz, M. C. *et al.* The subthalamic nucleus in Parkinson's disease: somatotopic organization and physiological characteristics. *Brain : a journal of neurology* **124**, 1777–90 (2001).

74. Romanelli, P. *et al.* Microelectrode recording revealing a somatotopic body map in the subthalamic nucleus in humans with Parkinson disease. *Journal of neurosurgery* **100**, 611–8 (2004).

75. Rossi, L. *et al.* Auditory and somatosensory evoked potentials (AEPs and SEPs) and ballistic movements in Parkinson disease. *Italian journal of neurological sciences* **6**, 329–37 (1985).

76. Rossini, P. M. *et al.* Abnormalities of short-latency somatosensory evoked potentials in parkinsonian patients. *Electroencephalography and clinical neurophysiology* **74**, 277–89 (1989).

77. Rossini, P. M., Bassetti, M. A. & Pasqualetti, P. Median nerve somatosensory evoked potentials. Apomorphine-induced transient potentiation of frontal components in Parkinson's disease and in parkinsonism. *Electroencephalography and clinical neurophysiology* **96**, 236–47 (1995).

78. Rossini, P. M. *et al.* Brain excitability and long latency muscular arm responses: non-invasive evaluation in healthy and parkinsonian subjects. *Electroencephalography and clinical neurophysiology* **81**, 454–65 (1991).

79. Rossini, P. M. *et al.* Parkinson's disease and somatosensory evoked potentials: apomorphine-induced transient potentiation of frontal components. *Neurology* **43**, 2495–500 (1993).

80. Rothblat, D. S. & Schneider, J. S. Alterations in pallidal neuronal responses to peripheral sensory and striatal stimulation in symptomatic and recovered parkinsonian cats. *Brain research* **705**, 1–14 (1995).

81. Sasaki, T. *et al.* Identification of Somatotopic Organization and Optimal Stimulation Site Within the Subthalamic Nucleus for Parkinson's Disease. *Operative neurosurgery (Hagerstown, Md.)* **17**, 239–246 (2019).

82. Schmiedt, C., Meistrowitz, A., Schwendemann, G., Herrmann, M. & Basar-Eroglu, C. Theta and alpha oscillations reflect differences in memory strategy and visual discrimination performance in patients with Parkinson's disease. *Neuroscience letters* **388**, 138–43 (2005).

83. Schrader, C. *et al.* Changes in processing of proprioceptive information in Parkinson's disease and multiple system atrophy. *Clinical Neurophysiology* **119**, 1139–46 (2008).

84. Seiss, E., Praamstra, P., Hesse, C. W. & Rickards, H. Proprioceptive sensory function in Parkinson's disease and Huntington's disease: evidence from proprioception-related EEG potentials. *Experimental brain research* **148**, 308–19 (2003).

85. Stefani, A. *et al.* Subdyskinetic apomorphine responses in globus pallidus and subthalamus of parkinsonian patients: lack of clear evidence for the 'indirect pathway'. *Clinical neurophysiology : official journal of the International Federation of Clinical Neurophysiology* **113**, 91–100 (2002).

86. Steigerwald, F. *et al.* Neuronal activity of the human subthalamic nucleus in the parkinsonian and nonparkinsonian state. *J Neurophysiol* **100**, 2515–2524 (2008).

87. Sterio, D. *et al.* Neurophysiological properties of pallidal neurons in Parkinson's disease. *Annals of neurology* **35**, 586–91 (1994).

88. Taha, J. M., Favre, J., Baumann, T. K. & Burchiel, K. J. Characteristics and somatotopic organization of kinesthetic cells in the globus pallidus of patients with Parkinson's disease. *Journal of neurosurgery* **85**, 1005–12 (1996).

89. Tamburin, S. *et al.* Abnormal sensorimotor integration is related to disease severity in Parkinson's disease: a TMS study. *Movement Disorders* **18**, 1316–24 (2003).

90. Theodosopoulos, P. V., Marks, W. J., Jr., Christine, C. & Starr, P. A. Locations of movement-related cells in the human subthalamic nucleus in Parkinson's disease. *Movement disorders : official journal of the Movement Disorder Society* **18**, 791–8 (2003).

91. Tinazzi, M., Fiaschi, A., Idone, D., Tezzon, F. & Zanette, G. Parkinson's disease and lower limb somatosensory evoked potentials: apomorphine-induced relief of the akinetic-rigid syndrome and vertex P37-N50 potentials. *Journal of the neurological sciences* **164**, 163–71 (1999).

92. Traversa, R. *et al.* N30 wave of somatosensory evoked potentials in Parkinson's disease: a

pharmacological approach. *Electroencephalography and clinical neurophysiology. Supplement* **46**, 193–200 (1996).

93. Trenado, C. *et al.* Long-Latency Somatosensory Evoked Potentials of the Subthalamic Nucleus in Patients with Parkinson's Disease. *PloS one* **12**, e0168151 (2017).
94. Tsai, S. T. *et al.* Dorsolateral subthalamic neuronal activity enhanced by median nerve stimulation characterizes Parkinson's disease during deep brain stimulation with general anesthesia. *Journal of neurosurgery* **123**, 1394–400 (2015).
95. Vinding, M. C. *et al.* Attenuated beta rebound to proprioceptive afferent feedback in Parkinson's disease. *Scientific reports* **9**, 2604 (2019).
96. Vyas, S., Huang, H., Gale, J. T., Sarma, S. V. & Montgomery, E. B. Neuronal Complexity in Subthalamic Nucleus is Reduced in Parkinson's Disease. *IEEE transactions on neural systems and rehabilitation engineering : a publication of the IEEE Engineering in Medicine and Biology Society* **24**, 36–45 (2016).
97. Weder, B. *et al.* Disturbed functional brain interactions underlying deficient tactile object discrimination in Parkinson's disease. *Human brain mapping* **11**, 131–45 (2000).
98. Weise, D. *et al.* Assessment of brainstem function with auricular branch of vagus nerve stimulation in Parkinson's disease. *PloS one* **10**, e0120786 (2015).
99. Yokoyama, T. *et al.* Neural activity of the subthalamic nucleus in Parkinson's disease patients. *Acta Neurochir (Wien)* **140**, 1287–1290; discussion 1291 (1998).
100. Zaidel, A., Spivak, A., Grieb, B., Bergman, H. & Israel, Z. Subthalamic span of beta oscillations predicts deep brain stimulation efficacy for patients with Parkinson's disease. *Brain : a journal of neurology* **133**, 2007–21 (2010).
101. Zhao, Y. *et al.* Altered activation in visual cortex: unusual functional magnetic resonance imaging finding in early Parkinson's disease. *The Journal of international medical research* **42**, 503–15 (2014).
